# Design Principles for Riboswitch Function

**DOI:** 10.1371/journal.pcbi.1000363

**Published:** 2009-04-17

**Authors:** Chase L. Beisel, Christina D. Smolke

**Affiliations:** Division of Chemistry and Chemical Engineering, California Institute of Technology, Pasadena, California, United States of America; Lawrence Berkeley National Laboratory, United States of America

## Abstract

Scientific and technological advances that enable the tuning of integrated regulatory components to match network and system requirements are critical to reliably control the function of biological systems. RNA provides a promising building block for the construction of tunable regulatory components based on its rich regulatory capacity and our current understanding of the sequence–function relationship. One prominent example of RNA-based regulatory components is riboswitches, genetic elements that mediate ligand control of gene expression through diverse regulatory mechanisms. While characterization of natural and synthetic riboswitches has revealed that riboswitch function can be modulated through sequence alteration, no quantitative frameworks exist to investigate or guide riboswitch tuning. Here, we combined mathematical modeling and experimental approaches to investigate the relationship between riboswitch function and performance. Model results demonstrated that the competition between reversible and irreversible rate constants dictates performance for different regulatory mechanisms. We also found that practical system restrictions, such as an upper limit on ligand concentration, can significantly alter the requirements for riboswitch performance, necessitating alternative tuning strategies. Previous experimental data for natural and synthetic riboswitches as well as experiments conducted in this work support model predictions. From our results, we developed a set of general design principles for synthetic riboswitches. Our results also provide a foundation from which to investigate how natural riboswitches are tuned to meet systems-level regulatory demands.

## Author Summary

Riboswitches are RNA-based components that integrate ligand binding and gene regulation to dynamically respond to molecular signals within cells. Natural riboswitches are employed to regulate metabolism and other cellular processes, while synthetic riboswitches have been constructed to expand the sensory and regulatory capabilities exhibited in nature. Characterization studies have revealed that sequence modifications can tune properties of the riboswitch response curve, which links ligand concentration to expression levels. Tunability is critical when matching component properties to the regulatory demands of biological systems; however, the characterization of riboswitch tuning strategies is complicated by the integration of numerous regulatory mechanisms and various processes, such as RNA folding and turnover, that impact riboswitch performance. To develop a generalized framework for examining quantitative aspects of riboswitch tuning, we modeled the kinetics of riboswitch function operating under common regulatory mechanisms. Our results reveal that riboswitch performance is primarily dictated by the competition between reversible and mechanism-specific irreversible rate constants. We demonstrate that practical system restrictions can significantly alter the requirements for riboswitch performance, necessitating a variety of tuning strategies. We developed design principles to guide the construction of synthetic riboswitches and a quantitative framework from which to investigate how natural riboswitches are tuned to meet systems-level regulatory demands.

## Introduction

The breadth of function exhibited by biological systems provides a foundation from which to develop solutions to global challenges including sustainability, renewable energy production, material synthesis, and medical advancement. Underlying these systems-level functions are regulatory components that evaluate molecular information in the extracellular and intracellular environments and ultimately translate that information into phenotypic responses over varying time scales. The properties of individual regulatory components and genetic networks composed of these components are tuned to control critical functions, including survival in fluctuating environments [1],[2], minimization of energy expenditure in metabolism [3],[4], developmental fate assignment [5], and proper information transmission through regulatory cascades [6]–[8]. To more effectively approach the reliable construction of synthetic biological systems, it is critical to advance our understanding of the degree to which individual component properties are tuned in natural systems, the underlying mechanisms that support tuning of biological components, and the effect of tuned components on resulting systems-level functions.

Riboswitches are RNA-based regulatory components that mediate ligand control of gene expression. Natural riboswitches have been identified in all three kingdoms of life [9] and primarily function by sensing a variety of essential cofactors, amino acids, and nucleotides and regulating the expression levels of proteins in associated metabolic pathways [10]. Riboswitches typically exploit three properties of RNA to translate changes in ligand concentration to changes in the expression of a target protein: specific and high affinity ligand binding by aptamer sequences, formation of distinct functional conformations primarily dictated by base-pairing interactions, and diverse gene expression regulatory mechanisms based on the central location of mRNA in the process of gene expression. With the exception of the *glmS* ribozyme [11],[12], natural riboswitches function through a general mechanism in which the RNA molecule can primarily adopt two conformations and ligand binding to the formed aptamer in one conformation biases partitioning toward the ligand-bound conformation. Each conformation is associated with differential regulatory activities such that increasing ligand concentrations either increase (ON behavior) or decrease (OFF behavior) gene expression depending on which conformation contains the formed aptamer. Synthetic riboswitches have been constructed based on this functional mechanism to expand on the regulatory potential exhibited by natural riboswitches [13],[14]. There has been significant interest in engineering riboswitches as tailored ligand-responsive genetic control elements by integrating aptamers selected *in vitro* [15] against diverse molecular ligands appropriate for different applications.

Natural and synthetic riboswitches have been demonstrated to be highly tunable regulatory components. Targeted nucleotide changes in synthetic riboswitches can shift the response curve [16]–[20]. Studies of natural riboswitches functioning through transcriptional termination found that the time lag between transcription of the aptamer and the terminator stem can tune the effective ligand concentration at which a half-maximal response is achieved (EC_50_) [21],[22]. These previous studies explored tuning in limited contexts by focusing on one aspect of riboswitch function (EC_50_) for one type of regulatory mechanism. However, advancing the characterization or design of new riboswitches requires a general quantitative framework that applies broadly to different regulatory mechanisms. Due to the link between RNA secondary structure and function and the relative ease with which RNA molecules can be modeled, riboswitches present an interesting class of regulatory components through which researchers can examine links between physical composition, tuned component response properties, and resulting systems-level behavior.

In this study, we employed mathematical modeling to explore how the dynamics of riboswitch function dictate its performance, where performance is evaluated based on the response curve quantitatively linking ligand concentration and protein levels. To draw general conclusions regarding riboswitch performance, we considered three representative regulatory mechanisms: transcriptional termination, translational repression, and mRNA destabilization. Parameter space for all three mechanisms was surveyed in order to understand the relationship between model parameters and riboswitch performance. Our results show that the competition between reversible and mechanism-specific irreversible rate constants primarily dictates riboswitch performance and response curve tuning properties. Complete dominance of irreversible rate constants renders a riboswitch non-functional, although ligand binding during transcription can rescue riboswitch performance. Our results also demonstrate that placing an upper limit on the ligand concentration alters the observed tuning properties such that a maximum dynamic range exists for intermediate conformational partitioning. Model predictions are supported by published experimental data and new data obtained through the modification of a synthetic riboswitch. We provide a set of design principles for the construction of synthetic riboswitches based on our modeling results. In addition, our results lend insights into the inherent flexibility and potential biological relevance of tuning of natural riboswitches.

## Results

### Kinetic modeling of riboswitch function

We started with a detailed molecular description of riboswitch function (Figure S1) that accounts for folding and ligand binding during discrete steps of transcription. Three regulatory mechanisms were considered: translational repression, transcriptional termination, and mRNA destabilization. Translational repression occurs through ribosome binding site (RBS) sequestration in a double-stranded secondary structure that prevents ribosome recruitment. Transcriptional termination occurs through a rho-independent mechanism such that hairpin formation directly upstream of a polyuridine stretch induces dissociation of the transcript from the template and the polymerase. We also considered the regulatory mechanism of a recently-described synthetic riboswitch that undergoes ribozyme self-cleavage [19], thereby initiating mRNA destabilization [23]. In these examples, two inter-converting conformations (A/B) are associated with differential protein levels subject to the specified regulatory mechanism. Ligand binding to the formed aptamer harbored in B promotes conformational stabilization, thereby increasing the prevalence of B.

We assigned a rate constant to each mechanistic step in the models to yield a detailed relationship between ligand concentration (L) and protein levels (P). In all models, transcriptional initiation produces a partial-length riboswitch in either conformation A (k_fA_) or B (k_fB_) to reflect transcriptional folding. Transcription is broken into discrete steps that represent different sequence lengths. Each step determines the extent of conformational switching (k_1_, k_1_′), the ability to bind and release ligand (k_2_, k_2_′), and the rate of progression to the next step (k_E_). For transcriptional termination, riboswitches effectively choose between termination (k_TA_, k_TB_) and extension (k_MA_, k_MB_) after transcription of the terminator stem. To ensure that both conformations make the decision with the same frequency, we set the sum of termination and extension rate constants for each conformation equal to a single parameter k_M_:
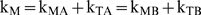
(1)Following transcription of the full-length riboswitch for translational repression and mRNA destabilization or extension through the terminator stem for transcriptional termination, the transcript can be translated into protein (k_PA_, k_PB_) or undergo degradation (k_dMA_, k_dMB_). A single constant is assigned when the rate constants are equal between conformations (k_P_, k_dM_). Values for the rate constants can vary widely depending on the organism and regulatory mechanism (Table 1). Therefore, we explored how each rate constant contributes to riboswitch performance.

**Table 1:** Estimated ranges for parameter values based on previous experimental and computational studies.

Parameter	Units	Value Range	References
k_f_	M/s	10^−13^–10^−8^	[46]
k_P_	1/s	10^−4^–10^1^	[46]
k_E_ α	1/s	10^−2^–10^2^	[22],[47]
k_1_, k_1_′	1/s	10^−3^–10^3^	[37], [48]–[50]
k_2_	1/M·s	10^3^–10^8^	[21],[22],[25],[33],[34],[51],[52]
k_2_′	1/s	10^−3^–10^1^	[21],[22],[34],[51],[52]
k_M_	1/s	10^−2^–10^−1^	[22],[53]
k_dM_ (norm)β	1/s	10^−5^–10^−2^	[54]–[57]
k_dM_ (rib)γ	1/s	10^−1^	[58]
k_dP_ β	1/s	10^−5^–10^−2^	[59],[60]

Rates for endogenous mRNA degradation (norm) and ribozyme cleavage (rib) are separately described.

**α:** Reflects the time to reach the termination stem following transcription initiation and is dependent on the rate of polymerase extension, pausing, the length of the transcribed sequence, and nucleotide concentration.

**β:** Includes mRNA or protein degradation and dilution due to cell division.

**γ:** Observed upper limit.

**Figure 1:**
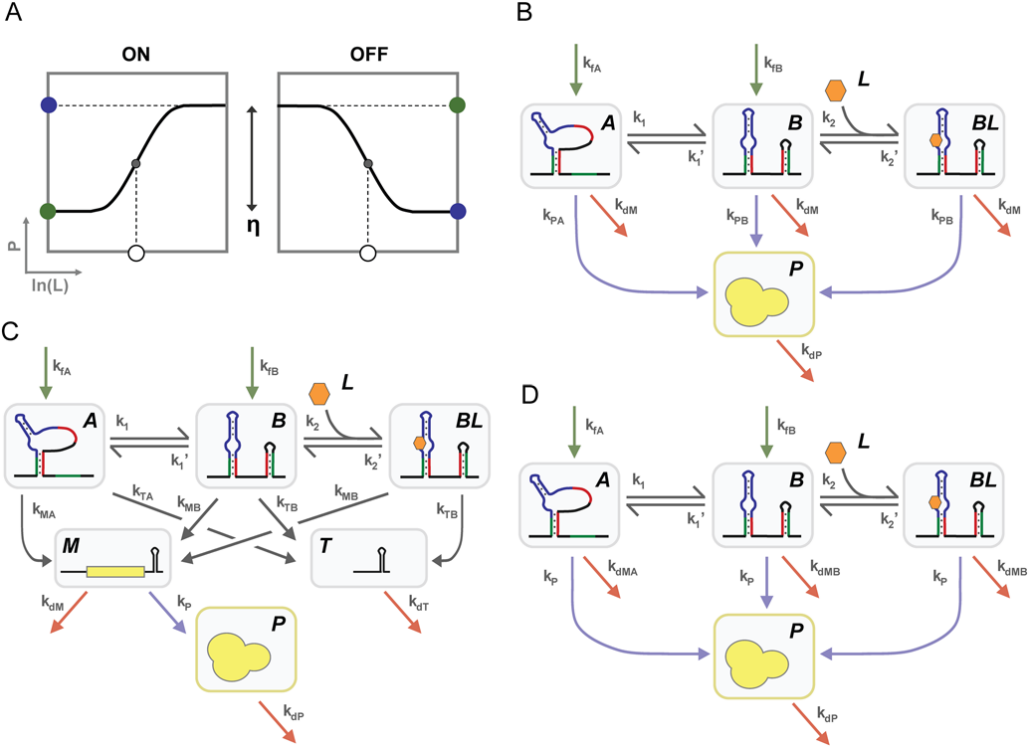
Kinetic models for riboswitches functioning through three distinct regulatory mechanisms. (A) General response curve relating ligand concentration (L) and regulated protein levels (P) for both ON and OFF behavior. Descriptors important in evaluating riboswitch performance are indicated: dynamic range (η), EC_50_ (○), basal protein levels (green •), and ligand-saturating protein levels (blue •). Gene regulatory mechanisms include (B) translational repression, (C) transcriptional termination, and (D) mRNA destabilization. All riboswitches can reversibly switch between conformations A and B that display different regulatory activities or different rates of degradation. Conformation B contains a formed aptamer that can reversibly bind ligand. Models assume negligible ligand binding during transcription. Green arrows designate mRNA synthesis with biased transcriptional folding, red arrows designate species degradation, and blue arrows designate translation that is proportional to mRNA levels. Under transcriptional termination (C), riboswitches effectively choose between termination to form a truncated product (T) and extension to form the full-length mRNA (M). To ensure both conformations make the decision with the same frequency, we designated a rate constant k_M_ equal to the sum of the rate constants for extension and termination for either conformation (k_M_ = k_MA_+k_TA_ = k_MB_+k_TB_).

Riboswitch performance was evaluated under steady-state conditions for both ON and OFF behaviors by calculating a collection of performance descriptors that define the response curve (Figure 1A): EC_50_, dynamic range (η) defined as the difference between high and low protein levels, basal protein levels (P(L = 0)), and ligand-saturating protein levels (P(L→∞)). While the dynamic range can be alternatively defined as the ratio of high and low protein levels, we selected the difference definition based on the mathematical symmetry between the equations representing ON and OFF behaviors (Text S1).

Transcription can be considered as a discrete multistep process (Figure S1). The conformations that can form at each step depend on the ordering of elements along the riboswitch sequence, such as the relative location of the aptamer or gene regulatory elements. Matching the number of steps and parameter values to particular sequence configurations becomes burdensome and restricts the elucidation of general principles. Therefore, we simplified the transcription process by assuming that synthesized transcripts appear in either conformation A or B and are immediately subject to conformational partitioning, ligand binding, and the regulatory mechanism (Figure 1B–D). As a result, the outcome of the transcription process is reflected by biased folding into either conformation A (k_fA_) or conformation B (k_fB_). This simplification excludes ligand binding during transcription, which has been demonstrated for natural riboswitches functioning through transcriptional termination [21],[22]. Therefore, we separately accounted for ligand binding during transcription in our analyses.

### Competition between reversible and irreversible rate constants suggests three operating regimes

We first derived expressions for the performance descriptors—dynamic range, EC_50_, and basal and ligand-saturating levels—for riboswitches functioning through each regulatory mechanism (Text S2). From these derivations two common parameters emerged: the partitioning constant (K_1_ = k_1_′/k_1_) and the ligand association constant (K_2_ = k_2_/k_2_′). K_1_ reflects the relative stability of conformation A and is present in all performance descriptor expressions. K_2_ reflects the affinity between the aptamer and its cognate ligand and is only present in the expression for EC_50_.

For all regulatory mechanisms, K_1_ and K_2_ reflect reversible conformational switching and ligand association, the core processes of riboswitch function. These processes are opposed by irreversible events that deplete the abundance of both conformations: mRNA degradation for translational repression and mRNA destabilization, and the riboswitch's decision to terminate or extend for transcriptional termination. The ratio between the rate constants for irreversible and reversible events is prevalent in all expressions for the performance descriptors (Text S2). This ratio is encapsulated in two reduced parameters γ_1_ and γ_2_:
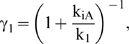
(2)

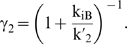
(3)Here, k_iA_ and k_iB_ represent the irreversible rate constants for conformation A or B, respectively, for translational repression (k_dM_), transcriptional termination (k_M_), and mRNA degradation (k_dMA_ and k_dMB_).

Three operating regimes can be defined based on the ratio of reversible and irreversible rate constants within γ_1_ and γ_2_. The first regime occurs when both reversible rate constants dominate (γ_1_, γ_2_ converge to one), the second begins when either of the reversible rate constants is balanced with the associated irreversible rate constant (either γ_1_ or γ_2_ is less than one), and the third begins when the irreversible rate constant k_iA_ dominates over k_1_ (γ_1_ converge to zero). Each regime is generally determined by the competition between reversible and irreversible rate constants. We next evaluated the tuning properties of each regime for all regulatory mechanisms.

### Riboswitches display similar tuning trends in the thermodynamically-driven regime

For dominating reversible rate constants (γ_1_ = γ_2_ = 1), a riboswitch molecule can sample both conformations and bind and unbind ligand many times before the irreversible event occurs. We define this regime as ‘thermodynamically-driven’ in accord with previous uses of this term in the study of natural riboswitches [21],[24],[25], since energetics dictate the prevalence of each conformation.

**Figure 2:**
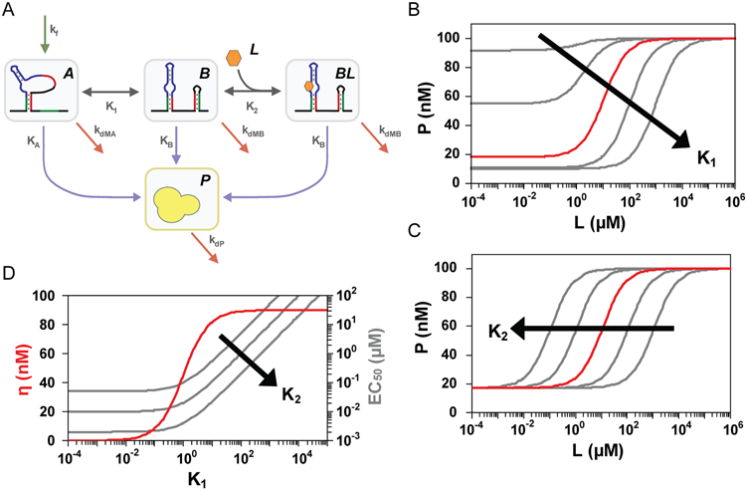
Thermodynamically-driven riboswitches display similar tuning properties. (A) Simplified molecular description that captures the three riboswitch regulatory mechanisms in the thermodynamically-driven regime. K_1_ is the conformational partitioning constant (k_1_′/k_1_) and K_2_ is the aptamer association constant (k_2_/k_2_′). Coloring is the same as in Figure 1B–D. K_A_ and K_B_ reflect the regulatory activity of conformations A and B, respectively, and are specific to each regulatory mechanism: translational repression (k_PA_, k_PB_), transcriptional termination (k_P_·k_MA_/k_M_, k_P_·k_MB_/k_M_), and mRNA destabilization (k_P_, k_P_). (B) K_1_ affects both basal levels and EC_50_. (C) K_2_ only affects EC_50_. Parameter values for red response curves: K_1_ = 10; K_2_ = 1/µM; K_A_ = 10^−3^/s; K_B_ = 10^−2^/s; k_f_ = 10^−11^ M/s; k_dP_ = 10^−3^/s; k_dMA_ = k_dMB_ = 10^−3^/s. (D) Biased conformational partitioning toward conformation B maximizes the dynamic range (η) at the cost of increased EC_50_.

In the thermodynamically-driven regime, riboswitch function is captured for the three regulatory mechanisms by a general molecular description (Figure 2A). The associated response curve is captured by a single equation that includes the partitioning constant (K_1_), the aptamer association constant (K_2_), mRNA degradation rate constants (k_dMA_, k_dMB_), and representative regulatory activities of conformations A (K_A_) and B (K_B_):
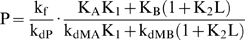
(4)The values of K_A_ and K_B_ depend on the selected regulatory mechanism and are provided in Text S2.

Parameter variation has a unique effect on the response curve for both ON (Figure 2B and 2C) and OFF behaviors (Figure S2). Increasing K_1_ stabilizes conformation A, resulting in more riboswitch molecules adopting this conformation. Since conformation A has lower regulatory activity for ON behavior (K_A_<K_B_), basal levels decrease. Concomitantly, EC_50_ increases as higher ligand concentrations are required to offset the decreased abundance of conformation B. Increasing K_2_ reduces EC_50_ as expected when aptamer affinity is modulated. However, K_2_ has no effect on dynamic range and ligand-saturating levels since we assumed sufficient ligand can be added to saturate the response curve. Previous mutational studies of two synthetic riboswitches [16],[17] support these model predictions. However, these studies examined trans-acting mechanisms, calling into question whether model insights apply to cis-acting mechanisms. Finally, rate constants distinct from the core processes of riboswitch function such as transcription initiation (k_f_) and protein decay (k_dP_) affect both basal levels and dynamic range by modulating the steady-state mRNA and protein levels.

Stabilizing conformation A (increasing K_1_) improves the dynamic range to an upper limit set by the regulatory activities (K_A_, K_B_) and mRNA degradation rate constants (k_dMA_, k_dMB_) associated with each conformation (Figure 2D). While all four parameters affect the dynamic range, k_dMA_ and k_dMB_ also impact the dependence of the dynamic range on conformational partitioning (Figure S3). This latter effect results from the dominant influence of the larger mRNA degradation rate on steady-state mRNA levels, which can be countered by biasing partitioning toward the more stable conformation. Therefore, when conformation A degrades faster (higher k_dMA_, ON behavior), less partitioning toward conformation A (lower K_1_) is required to separate basal and ligand-saturating levels, whereas more partitioning toward conformation A (higher K_1_) is required when conformation B degrades faster (higher k_dMB_, OFF behavior). As a result, thermodynamically-driven riboswitches functioning through mRNA destabilization require more (OFF behavior) or less (ON behavior) partitioning toward conformation A to achieve a larger dynamic range. In contrast, riboswitches functioning through translational repression and transcriptional termination display similar trends in dynamic range as a function of conformational partitioning for ON and OFF behaviors as the degradation rate constants are the same for each conformation.

EC_50_ is also dependent on the value of K_1_ according to the following relationship:
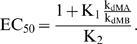
(5)EC_50_ approaches the lower limit set by the aptamer dissociation constant when riboswitches principally adopt conformation B (low K_1_). Therefore, although stabilizing conformation A (increasing K_1_) can improve the dynamic range, excessive stabilization can be detrimental due to the increase in EC_50_. As a result, tuning strategies based on increasing K_1_ require higher ligand concentrations to access the improved dynamic range. The ratio of the mRNA degradation rate constants in the expression for EC_50_ offsets the modified dependence of the dynamic range on K_1_ for riboswitches functioning through mRNA destabilization. Therefore, riboswitches functioning through any of the regulatory mechanisms exhibit the same trade-off between EC_50_ and dynamic range.

### Riboswitches display expanded tunability with reduced performance in the kinetically-driven regime

**Figure 3:**
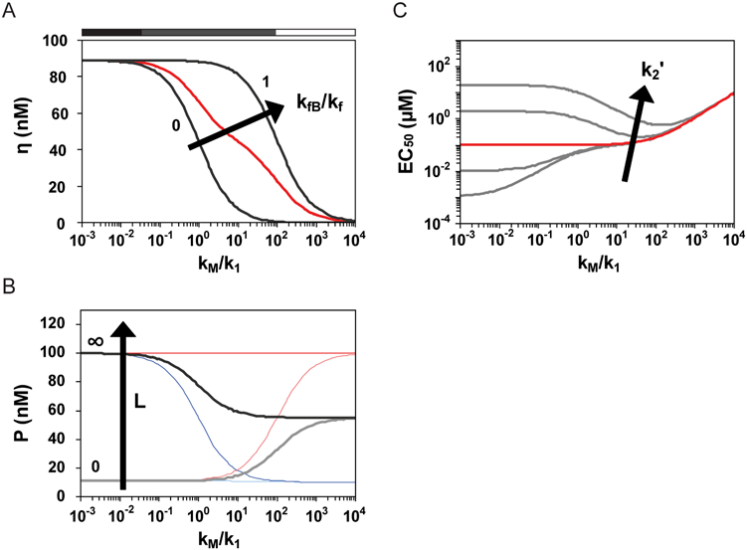
Rate competition dictates riboswitch performance. The relative values of the reversible and irreversible rate constants generally establish three operating regimes: thermodynamically-driven (▪) when reversible rate constants dominate, kinetically-driven (▪) when the rate constants are balanced, and non-functional (□) when irreversible rate constants dominate. Regimes are qualitatively marked for dynamic range and basal and ligand-saturating levels according the ratio of the rate constants for terminator stem formation (k_M_) and the progression from conformation A to conformation B (k_1_). Effect of varying k_M_ on (A) dynamic range, (B) basal protein levels and ligand-saturating protein levels, and (C) EC_50_ for riboswitches functioning through transcriptional termination. In (B), colored pairs show basal (light) and ligand-saturating (dark) protein levels for complete (red), balanced (black), and negligible (blue) transcriptional folding into conformation B. Parameter values for all curves in (A) and (B) and the red curve in (C): k_1_ = 10^−1^/s;k_1_′ = 10/s; k_2_ = 10^6^/M·s; k_2_′ = 10^−1^/s; K_A_ = k_P_·k_MA_/k_M_ = 10^−3^/s; K_B_ = k_P_·k_MB_/k_M_ = 10^−2^/s; k_f_ = 10^−11^ M/s; k_dP_ = 10^−3^/s.

The second regime begins when either of the irreversible rate constants balances the associated reversible rate constant (either γ_1_ or γ_2_ is between zero and one). We call this regime the ‘kinetically-driven’ regime in accord with uses of this term in the study of natural riboswitches [21],[22], where performance is driven by kinetics over energetics. In this regime, riboswitch molecules have fewer opportunities to sample both conformations and bind and release ligand before the irreversible event occurs, where the number of opportunities is governed by the competition between reversible and irreversible rate constants. Since γ_1_ is coupled to K_1_ and k_fA_ while γ_2_ is coupled to K_2_, both γ_1_ and γ_2_ are anticipated to have a significant impact on the response curve and impart several tuning properties distinct to this regime. We initially use riboswitches functioning through transcriptional termination to highlight two of these tuning properties (Figure 3A–C).

First, irreversible rate constants modulate all performance descriptors, often at a cost to riboswitch performance. As the rate constant for terminator stem formation (k_M_) increases, riboswitch molecules become trapped in a given conformation after transcriptional folding or conformational switching as reflected in γ_1_. This effect reduces the dynamic range (Figure 3A) and shifts basal and ligand-saturating levels according to the extent of transcriptional folding (Figure 3B). The reduction in dynamic range can be offset by increasing the overall mRNA and protein abundance through modulation of the rates of transcription (k_f_), translation (k_P_), and mRNA (k_dM_) and protein (k_dP_) degradation. However, such changes also increase basal levels.

γ_1_ and γ_2_ both impact EC_50_ according to the following relationship:
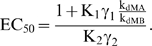
(6)Since γ_1_ and γ_2_ reflect the ratios of k_M_/k_1_ and k_M_/k_2_′, respectively, the relationship between EC_50_ and k_M_ depends on both conformational switching (k_1_) and ligand release (k_2_′) (Figure 3C). Increasing k_M_, which reduces both γ_1_ and γ_2_, can increase or decrease EC_50_ based on the opposing contributions of γ_1_ and γ_2_. Reducing γ_1_ decreases EC_50_ by restricting the time available to switch between conformations, while reducing γ_2_ increases EC_50_ by decreasing the half-life of the ligand-aptamer complex (BL). Therefore, the relative values of k_1_ and k_2_′ must be known to predict the effect of modulating the irreversible rate constant on EC_50_.

Second, biased transcriptional folding can modulate the relationship between irreversible rate constants and the dynamic range (Figure 3A). When transcriptional folding is biased toward conformation A (k_fB_/k_f_ = 0), riboswitch molecules must have sufficient time to switch between conformations to maintain activity. Therefore, the dynamic range declines as k_M_ approaches and surpasses k_1_. In contrast, when transcriptional folding is biased toward conformation B (k_fB_/k_f_ = 1), riboswitch molecules must switch to conformation A before the irreversible event occurs. In this case, k_M_ must exceed the sum 2k_1_+k_1_′ to reduce the dynamic range. As a result, biasing transcriptional folding toward conformation B in the kinetically-driven regime increases the dynamic range.

A third tuning property is associated with riboswitches functioning through mRNA degradation. The rate constants for mRNA degradation (k_dMA_, k_dMB_) are responsible for both the irreversible event and the steady-state basal and ligand-saturating levels, resulting in complex tuning properties (Figure S4). Increasing either k_dMA_ (ON behavior) or k_dMB_ (OFF behavior) initially improves the dynamic range by separating the steady-state basal and ligand-saturating levels. However, the impact of larger values of k_dMA_ and k_dMB_ depends on riboswitch behavior. For ON behavior, if a riboswitch predominantly folds into conformation A during transcription (k_fB_/k_f_ = 0), then values of k_dMA_ in excess of k_1_ diminish the dynamic range as conformation A is degraded before it can switch conformations. However, if a riboswitch predominantly folds into conformation B during transcription (k_fB_/k_f_ = 1), then the dynamic range plateaus as each molecule either binds ligand or irreversibly switches to conformation A. In contrast, transcriptional folding has a negligible impact on the relationship between the dynamic range and k_dMB_ for OFF behavior, since molecules that adopt conformation A will switch to conformation B before undergoing degradation. Furthermore, as observed for the thermodynamically-driven regime, more partitioning toward conformation A (higher K_1_) is required to counteract the influence of k_dMB_ on mRNA steady-state levels. Increasing k_dMB_ eventually dominates basal levels when partitioning is maintained, leading to a loss in the dynamic range (Figure S3).

As such, a tailored design approach is required to account for the difference between ON and OFF behavior for kinetically-driven riboswitches functioning through mRNA destabilization. Transcriptional folding is a key tuning parameter for ON behavior and should be the predominant focus before tuning the degradation rate of conformation A. In contrast, transcriptional folding can be largely ignored for OFF behavior, and the degradation rate of conformation B must be properly tuned to optimize the dynamic range.

### Rescuing riboswitch performance in the non-functional regime

Higher irreversible rate constants require increased ligand concentrations to achieve a diminishing change in protein levels. We define the ‘non-functional’ regime as one in which riboswitches are effectively trapped in the conformation formed during transcriptional folding (γ_1_ = 0). In this regime, ligand has a negligible effect on performance. The fast time scales of terminator stem formation and mRNA cleavage may drive riboswitches functioning through these regulatory mechanisms into this regime.

Our analysis of the kinetically-driven regime revealed that performance can be preserved by biasing transcriptional folding toward conformation B and ensuring that k_1_′ exceeds the irreversible rate constant k_iA_. However, these approaches do not alleviate the increased EC_50_ caused by the reduced half-life of the ligand-aptamer complex (BL) when γ_2_ approaches 0. As a potential solution, studies of natural riboswitches have suggested that ligand binding during transcription can preserve EC_50_ [21],[22]. Therefore, we examined the effect of ligand binding during transcription under the assumption that conformation B is solely available (k_fB_/k_f_ = 1) prior to polymerase extension (k_E_) through the gene regulatory element responsible for the irreversible event.

**Figure 4:**
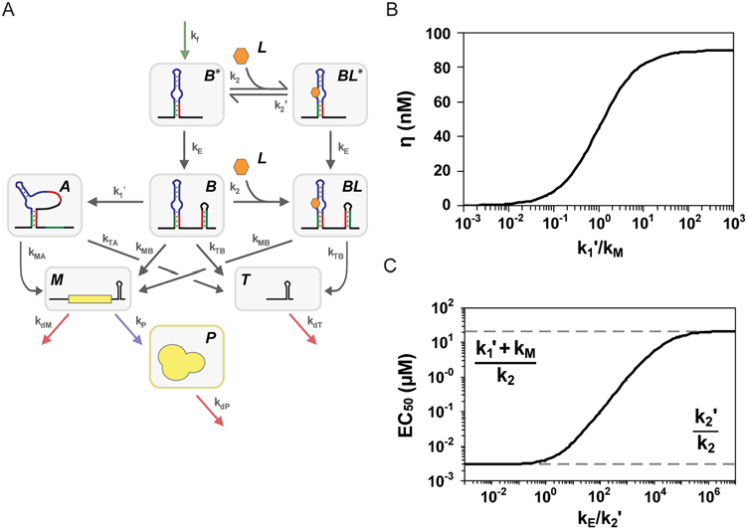
Rescuing riboswitch performance in the non-functional regime. (A) Molecular description of a non-functional riboswitch functioning through transcriptional termination. The description is identical to that in Figure 1C with notable exceptions: the aptamer in conformation B is transcribed first (B*) and can reversibly bind and release ligand before the terminator stem is transcribed (k_E_), and terminator stem formation (k_M_ = k_MA_+k_TA_ = k_MB_+k_TB_) occurs much faster than ligand release (k_2_′) and the progression from conformation A to B (k_1_). (B) The competition between terminator stem formation (k_M_) and the progression from conformation B to A (k_1_′) determines the dynamic range. (C) EC_50_ can be tuned independent from the dynamic range. The accessible range of EC_50_ values is bounded by the aptamer association constant (K_2_ = k_2_/k_2_′), the rate constant for the progression from conformation B to A (k_1_′), and the rate constant for terminator stem formation (k_M_). EC_50_ is tuned over this range by the rate constant representing the delay between aptamer formation and transcription of the terminator stem (k_E_). Parameter values: k_2_ = 10^6^/M·s; k_2_′ = 3·10^−3^/s; K_A_ = k_P_·k_MA_/k_M_ = 10^−3^/s; K_B_ = k_P_·k_MB_/k_M_ = 10^−2^/s; k_f_ = 10^−11^ M/s; k_dP_ = 10^−3^/s; k_dM_ = 10^−3^/s; k_1_′+k_M_ = 20/s.

We examined ligand binding during transcription for riboswitches functioning through transcriptional termination (Figure 4A). We assumed that terminator stem formation (k_M_) occurs much faster than ligand release (k_2_′) and the progression from conformation A to B (k_1_) to limit consideration to non-functional riboswitches. Under these assumptions, the dynamic range is dependent on the ratio of read-through efficiencies for conformations A (k_MA_/k_M_) and B (k_MB_/k_M_), the progression from conformation B to A (k_1_′), and the rate of terminator stem formation (k_M_). The dynamic range is maximized when conformational progression occurs much faster than terminator stem formation (Figure 4B) as predicted from our analysis of the kinetically-driven regime (Figure 3A). An *in vitro* study of the *ribD* FMN riboswitch operating through transcriptional termination yielded a reduced dynamic range when removing the polymerase pause site in the terminator sequence, increasing the nucleotide concentration, and withholding the NusA protein responsible for increasing polymerase residence time at pause sites [22]. These manipulations are expected to reflect an increase in k_M_ and thus support our model predictions. If increasing k_1_′ above k_M_ maximizes the dynamic range, riboswitches operating in this regime are expected to display strong stabilization of conformation A reflecting rapid progression from conformation B. In support of this claim, full-length riboswitches operating under transcriptional termination strongly prefer the aptamer-disrupted conformation and exhibit negligible ligand binding affinity [22],[25],[26].

EC_50_ tuning properties are strikingly different for riboswitches in which ligand binding during transcription allows for improved performance than those for thermodynamically-driven riboswitches. EC_50_ depends on model parameters in Figure 4A according to the following relationship:
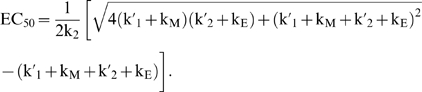
(6)Both ligand release (k_2_′) and the time necessary to transcribe the sequences required for the formation of conformation A (k_E_) have a significant impact on the value of EC_50_ (Figure 4C). Interestingly, tuning of k_E_ decouples EC_50_ and basal levels such that EC_50_ can equal the aptamer dissociation constant (k_2_/k_2_′) without impacting the dynamic range. In contrast, the EC_50_ of a thermodynamically-driven riboswitch approaches the aptamer dissociation constant as conformation B is stabilized, resulting in a concomitant decrease in the dynamic range (Figure 2D). A previous theoretical study of the *pbuE* adenine riboswitch using experimentally measured kinetic rates also concluded that modulating polymerase extension time can tune EC_50_ when the extension time is not significantly slower than ligand release [21].

### Restricting the ligand concentration upper limit alters observed tuning properties

**Figure 5:**
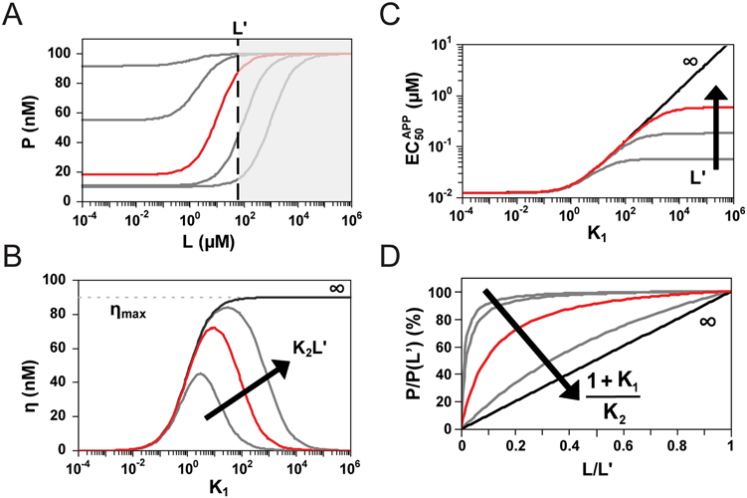
Placing an upper limit on the ligand concentration range alters the observed tuning properties. (A) Placing an upper limit on the ligand concentration (L') restricts access to the full response curve. This limit affects the dependence of (B) the dynamic range (η) and (C) the apparent EC_50_ (EC_50_^APP^) on the conformational partitioning constant (K_1_) and the aptamer association constant (K_2_) as demonstrated for a thermodynamically-driven riboswitch. The maximum dynamic range (η_max_) is proportional to the difference between regulatory activities for conformations A (K_A_) and B (K_B_) normalized to the respective degradation rate constants k_dMA_ and k_dMB_. (D) Normalized response curves for fixed L' and increasing values of (1+K_1_)/K_2_, which equals EC_50_ under ligand-saturating conditions. Parameter values are identical to those reported in Figure 2 with L' = 60 µM.

In our analyses thus far, we assumed that the maximum ligand concentration always saturates the response curve. However, studies of synthetic riboswitches have demonstrated that the response curve may not be saturated by the accessible upper limit in ligand concentration (Figure 5A) due to various system properties including aptamer affinity, ligand solubility, permeability of the ligand across the cell membrane, and cytotoxicity of the ligand [16], [17], [19], [27]–[29]. Furthermore, natural riboswitches may regularly function in response to physiologically-relevant changes in metabolite concentrations that are much smaller than the ∼1000-fold range necessary to access the full riboswitch response curve. To assess the effect of establishing an upper limit to the ligand concentration, we evaluated the response curve descriptors for a maximum ligand concentration of L'. An apparent EC_50_ (EC_50_^APP^) was calculated according to protein levels at L = 0 and L'.

Restricting L' alters the dependence of the dynamic range (Figure 5B) and the apparent EC_50_ (Figure 5C) on model parameters as illustrated for riboswitches operating in the thermodynamically-driven regime. L' acts as a system restriction that prevents access to the full response curve such that increasing K_1_ shifts the actual EC_50_ beyond L', thereby reducing the maximum dynamic range that can be achieved. This behavior was recently observed for a trans-acting synthetic riboswitch operating under a limited ligand concentration range [17], supporting model predictions. Reflecting this behavior, the apparent EC_50_ has the following dependence:
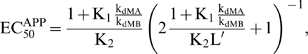
(7)where the apparent EC_50_ converges to L'/2 as expected for a linear response when L' is below the EC_50_ for an unbounded ligand concentration range (Figure 5D). Our modeling results demonstrate that restricting the ligand concentration upper limit reduces riboswitch performance and establishes a unique relationship between dynamic range and conformational partitioning. In addition to serving as a design constraint for synthetic riboswitches, natural riboswitches may inherently operate under defined limits in ligand concentration. Future experiments may focus on measuring the physiologically-relevant metabolite concentration range experienced by natural riboswitches to examine what section of the response curve is utilized.

### Application of tuning strategies to a synthetic riboswitch supports model predictions

**Figure 6:**
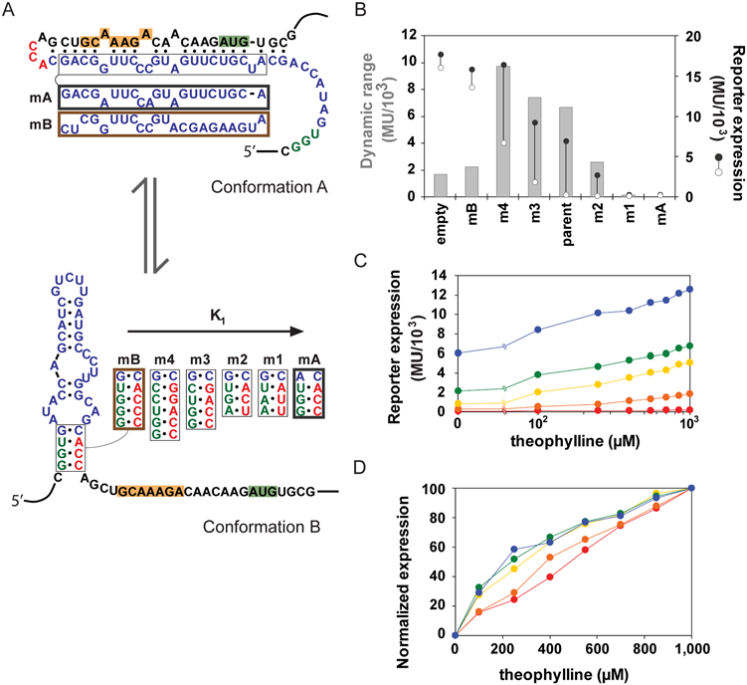
Mutational analysis of a synthetic riboswitch supports model predictions. (A) Mutations made to the aptamer stem of the parent synthetic riboswitch (m1–4) are anticipated to solely modulate conformational partitioning (K_1_). The theophylline-responsive riboswitch controls Tn10-β-Galactosidase levels through RBS sequestration, thereby repressing translation. Mutations were also introduced to lock the riboswitch in either conformation A (mA) or conformation B (mB). The RBS and start codon are highlighted in orange and green, respectively. (B) β-Galactosidase assay results are reported in Miller Units (MU) for each riboswitch variant in the presence (•) or absence (○) of 1 mM theophylline. Dynamic range (η) is calculated as the difference between high and low expression levels, where all values were below the theoretical maximum of 15,600 MU as determined by the difference between mB with theophylline and mA without theophylline. The positive control construct (empty) harbors only the RBS and aptamer basal stem. A slight increase in β-Galactosidase activity was observed in the presence of theophylline for the control construct. (C,D) Theophylline response curves for riboswitch variants: parent (yellow), m1 (red), m2 (orange), m3 (green), and m4 (blue). (C) Raw data and (D) normalized data illustrate the predicted shift in both basal levels and EC_50_ for increasing stabilization of conformation B. Data represent independent measurements of triplicate samples, where the standard error was below 5% of each mean value.

To begin evaluating how the predicted tuning trends apply to both natural and synthetic riboswitches, we physically manipulated a recently-described synthetic riboswitch functioning through translational repression that up-regulates gene expression (ON behavior) in the presence of theophylline [18] (Figure 6A). Under the naming convention from Figure 1B, conformation A comprises a base-paired structure between the aptamer and RBS, while conformation B includes a formed aptamer and a single-stranded RBS. This riboswitch was selected because it closely resembles natural riboswitches functioning through translational repression, experimental data suggest that this riboswitch operates in the thermodynamically-driven regime [18], the ligand concentration upper limit does not saturate the response curve [28], and the demonstration that different sequences yield different response curves suggests riboswitch tuning [18]. A theophylline concentration of 1 mM was used as an upper limit, as exceeding this concentration inhibited cell growth. In studies performed by Lynch and coworkers, sequences associated with desirable response curves were identified by randomization of the RBS and screening for variants with low basal activity and a large activity increase in the presence of theophylline. Since the randomized sequence was located in a region responsible for conformational partitioning and translation, mutations most likely reflect simultaneous modulation of K_A_, K_B_, and K_1_. We therefore sought to introduce directed mutations to solely modulate individual model parameters and test model predictions for a thermodynamically-driven riboswitch with a ligand concentration upper limit that prevents response curve saturation.

We examined two model predictions that could not be supported with available experimental data for cis-acting riboswitches: (1) solely modulating conformational partitioning (K_1_) affects both EC_50_ and basal levels (Figure 2B), and (2) the dynamic range can be optimized by modulating K_1_ when the ligand concentration upper limit does not saturate the response curve (Figure 5B). We modulated K_1_ by introducing systematic mutations into the aptamer stem while preserving the RBS sequence (m1–4; Figure 6A). Mutant sequences were ordered with increasing K_1_ based on the energetic difference between conformations predicted by the RNA folding algorithm Mfold [17]. The mutations were not anticipated to significantly affect aptamer affinity (K_2_) [30],[31] or translational efficiency for either conformation (K_A_, K_B_). Additional mutants were examined that are predicted to entirely favor either conformation A (mA) or conformation B (mB) to establish the regulatory activity of either conformation. Riboswitch performance was evaluated by measuring β-Galactosidase levels over a range of theophylline concentrations.

The introduced mutations altered the response curve in agreement with model predictions (Figure 6B–D). Protein levels in the presence and absence of theophylline correlated with the relative stability of conformation A. Furthermore, complete stabilization of conformation A (mA) and conformation B (mB) established respective lower and upper limits for the observed expression levels. As predicted for a non-saturating value of L', an intermediate conformational partitioning value optimized the dynamic range to a value that was below the maximum dynamic range (η_max_ = 15,600 MU) (Figure 6B), and EC_50_ approached 0.5 mM (L'/2) for increased stabilization of conformation A (Figure 6C and 6D). Dynamic range optimization is clearly observed when evaluating the ratio of high and low protein levels, which is predicted to display the same qualitative tuning behavior (Figure S5). The data agree with our model predictions for K_1_ modulation in the thermodynamically-driven regime under conditions where the ligand concentration upper limit does not saturate the response curve, although we cannot rule out the possibility that stabilization of conformation A inadvertently drove the riboswitch into the kinetically-driven regime. The introduction of the aptamer sequence to the regulatory element decreased the regulatory activity of conformation B as observed when comparing protein levels for mB and a construct harboring only the RBS and aptamer basal stem (empty; Figure 6B). Our previous construction and characterization of a trans-acting synthetic riboswitch functioning through RNA interference [17] also showed sub-maximum dynamic range optimization when the ligand concentration was limiting and compromised activity of the regulatory element due to introduction of the aptamer element of the riboswitch. Thus, the results support the extension of our model predictions to synthetic riboswitches. In addition, our modeling results may have direct implications for the performance and tuning of natural riboswitches based on the similarity between the synthetic riboswitch examined here and natural riboswitches operating under translational repression.

## Discussion

While our modeling efforts focused on translational repression, transcriptional termination, and mRNA destabilization, the predicted tuning trends generally apply to riboswitches utilizing other regulatory mechanisms. For example, riboswitches that function through alternative splicing mechanisms [32] can be modeled using the same approach applied to transcriptional termination-based riboswitches, where the splicing event occurs during a time interval in which riboswitch conformation determines the final exon composition. The identity and relative value of the irreversible rate constant for each conformation are important in determining the tuning properties associated with different regulatory mechanisms. The varying effects of irreversible rate constants on riboswitch performance are highlighted by the different tuning properties for riboswitches functioning through transcriptional termination and mRNA destabilization in the kinetically-driven regime.

The competition between reversible and irreversible rate constants establishes three operating regimes with distinct tuning properties. Therefore, measuring the reversible and irreversible rate constants is critical when predicting the impact of parameter modulation on the response curve. While well-established methods allow measurement of the rates of mRNA degradation and ligand binding and release, measuring the rates of RNA folding and conformational inter-conversion is currently an active area of research. New technologies are emerging that allow the measurement of kinetic folding rates: site-specific incorporation of aminopurines [25],[33], single-molecule force experiments [34]–[36], and single-molecule fluorescence resonance energy transfer [37]. Studies of natural and synthetic riboswitches that apply these approaches may yield a comprehensive understanding of the relationship between riboswitch function and performance [38].

An alternative approach to measuring conformational switching relies on parameter predictions with RNA folding algorithms. Most algorithms calculate the free energy of individual conformations and can be used to estimate the value of K_1_ for a riboswitch sequence [39],[40]. Algorithms have also been developed that provide estimates of the rate constants for conformational switching (k_1_, k_1_′) [41]. By employing these algorithms, sequences can be rapidly screened *in silico* to identify riboswitches with tuned conformational partitioning according to model predictions. Mutations that impact other parameters, such as mutations to the RBS sequence that affect regulatory activity, can also be screened *in silico* to evaluate the impact on secondary structure and conformational partitioning. However, these algorithms are often inaccurate when predicting RNA folding *in vivo*, requiring modified approaches [17] or the development of more advanced algorithms [40].

### Design principles for synthetic riboswitches

Synthetic riboswitches can be divided into two categories based on the intended application: inducible regulators and autonomous regulators. The applicable category depends on the identity and source of the detected ligand and requires distinct approaches to riboswitch design. We provide the following design principles assembled from our modeling results to guide the design of synthetic riboswitches as inducible or autonomous regulatory systems.

The desired properties of inducible regulatory systems include large dynamic ranges, low basal expression levels, and titratable control over expression levels. Selecting an effective regulatory mechanism is critical since numerous factors reduce the dynamic range, such as conformational partitioning, dominating irreversible rates, upper limits to ligand concentration, and reduced gene regulatory efficiencies from the incorporation of other riboswitch elements [17],[19]. A design that is biased toward forming the disrupted-aptamer conformation (high K_1_) will generally increase the dynamic range, although such strategies require higher ligand concentrations to modulate protein levels. The rates of events separate from core riboswitch processes, such as transcription, translation, and protein decay, can be modulated to increase the dynamic range difference at the expense of increased basal levels.

The selected regulatory mechanism will likely dictate the values of the irreversible rate constants and thus the operating regime. In support of this, studies on natural riboswitches have suggested a consistent pairing between translational repression and the thermodynamically-driven regime [25] and transcriptional termination and the non-functional regime with ligand binding during transcription [21],[22],[25],[26],[33]. Therefore, the design of inducible regulatory systems may rely on the tuning properties associated with each regime. While thermodynamically-driven riboswitches generally provide for the largest dynamic range, kinetically-driven and non-functional riboswitches can be designed to perform similarly using insights from our modeling efforts. In general, placing the aptamer toward the 5′ end of the riboswitch sequence will preserve the dynamic range by biasing transcriptional folding toward conformation B. The exception is OFF-behaving riboswitches acting through mRNA destabilization, which are insensitive to biased transcriptional folding (Figure S4). In addition, introducing pause sites and ensuring rapid conformational switching from the aptamer-formed conformation (k_1_′) will allow kinetically-driven and non-functional riboswitches to exploit ligand binding during transcription, thereby decreasing the amount of ligand required to induce gene expression.

**Figure 7:**
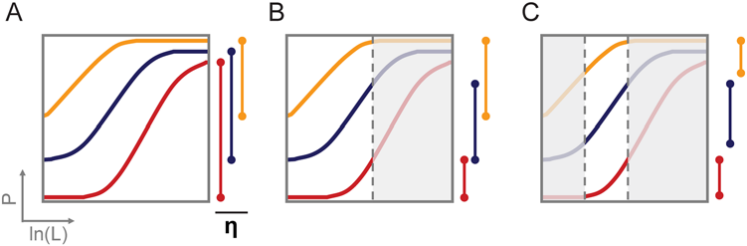
The accessibility of the riboswitch response curve depends on application category and associated system restrictions. Categories include an inducible regulatory system with (A) no ligand limitations or (B) a ligand concentration upper limit, and (C) an autonomous regulatory system with defined lower and upper limits for the ligand concentration. The accessible dynamic range (η) for each response curve depends on the system restrictions. The properties of other components in the network will dictate which riboswitch design best meets performance requirements. For example, under the autonomous regulatory system (C) the red curve may be more appropriate for the regulation of cytotoxic genes, the orange curve may be more appropriate for the regulation of enzymes with low catalytic activity, and the blue curve may be more appropriate for regulatory networks that require a large change in protein levels.

In many practical applications, system restrictions will limit the accessible range of the response curve (Figure 7A–C). Such limitations need to be addressed through parameter tuning in order to access the appropriate section of the response curve. For most biological systems, a predominant restriction is a limit to the maximum ligand concentration. In situations where the maximum ligand concentration does not saturate the response curve, designs for thermodynamically-driven riboswitches should be based on intermediate conformational partitioning values (K_1_) that achieve a suboptimal maximum dynamic range. An alternative strategy is the design of non-functional riboswitches that bind ligand during transcription, which can respond to ligand at lower concentrations without sacrificing the dynamic range.

Genes often exist in regulatory networks that dictate cellular phenotype such that complex relationships exist between the expression levels of individual genes and systems-level functions. To effectively regulate these genes with synthetic riboswitches, a variety of tuning strategies must be employed to tune the response curve to operate within system restrictions. The properties of the regulated gene, its integration into a biological network, and the ultimate systems-level functions must be considered. One can envision an application-specific regulatory niche that defines the acceptable ranges of basal and maximum-ligand expression levels for proper system performance (Figure 7B). For example, the properties of a given system may require that the riboswitch be tuned to minimize basal expression over maximizing dynamic range, such as when the regulated enzyme exhibits high activity or cytotoxicity.

The engineering of synthetic riboswitches that act as autonomous regulatory systems presents an even greater design challenge. Here, the upper and lower ligand concentrations that the system fluctuates between establish the accessible section of the response curve such that the regulatory niche is further restricted (Figure 7C). For example, riboswitches responsive to an endogenous central metabolite will likely be operating under a defined concentration range characteristic of the organism and the environment. In this case, the response curve must be tuned to place the desired expression levels at the limits of this defined concentration range by modulating the appropriate performance descriptors. Depending on system restrictions, proper tuning of riboswitches acting as autonomous control systems may require minimization of basal levels, operation across higher expression levels, or maximization of the change in expression levels.

Many parameters can potentially be modulated to tune the response curve. However, current practical considerations favor the modulation of a subset of these parameters in the laboratory. As one example, a given riboswitch may require a higher EC_50_ value to meet the performance requirements. Aptamer affinity (K_2_), conformational partitioning (K_1_), and the irreversible rates associated with the gene regulatory mechanism can be modulated to increase EC_50_. However, rational modulation of aptamer affinity is restrictive since most mutations effectively abolish ligand binding, while the method and ease of modulating irreversible rates depend on the regulatory mechanism. Modulating conformational partitioning is an attractive approach since simple base-pairing interactions principally establish each conformation. However, conformational partitioning also impacts basal levels and the dynamic range, such that other parameters may need to be modulated to compensate for any undesired changes. Thus, the effective design of synthetic riboswitches requires knowledge of the relationship between riboswitch sequence and model parameters and may require the coordinated modulation of multiple parameters to meet application-specific performance requirements.

The relationship between riboswitch sequence and model parameters depends in part on the composition framework used in the riboswitch design. A synthetic riboswitch can be designed such that parameters map to individual domains [16],[17],[19] or multiple domains [18],[42],[43]. Each design strategy offers distinct advantages depending on whether rational design or random screening is used to select riboswitch sequences. Individual domain mapping strategies allow for insulated control over each parameter and domain swapping without requiring redesign of the riboswitch, thereby presenting significant advantages for rational design approaches. Multiple domain mapping strategies may be more desirable for random screening approaches, where assigning multiple parameters to a single sequence domain can reduce the number of randomized nucleotides required to sufficiently sample parameter space.

### Evolutionary implications for tuning in natural riboswitches

Natural riboswitches primarily serve as key autonomous regulators of diverse metabolic processes [10]. Recent characterization of eleven known S-adenosylmethionine riboswitches in *Bacillus subtilis* demonstrated that these riboswitches exhibit a diverse range of values for basal expression levels, EC_50_, and dynamic range [44], suggesting that natural riboswitches are finely tuned to match their occupied regulatory niche. However, this study is the only one to date to characterize the response curves of multiple natural riboswitches responsive to the same ligand. Two questions emerge from these observations and our modeling results that underlie the biological utilization of natural riboswitches as dynamic regulators of metabolism: (1) how finely tuned are natural riboswitches to their regulatory niche, and (2) what sequence modifications are associated with response curve tuning?

Understanding the extent to which natural riboswitches are tuned to their regulatory niches will provide insights into riboswitch utilization and the underlying principles of genetic regulatory control. Similar to the tuning of synthetic riboswitches to match their intended regulatory niche, investigating the extent and biological relevance of natural riboswitch tuning requires knowledge of the functional properties of the regulated genes and their contribution to cellular fitness. Furthermore, the typical ligand concentration range encountered in the intracellular environment designates the operational section of the response curve, such that determining this range is critical to advancing our understanding of natural riboswitch tuning within regulatory niches.

The composition of a natural riboswitch dictates the relationship between its sequence and model parameters. One way to gain insights into this relationship is investigating sequence deviations between natural riboswitches in the same organism or different organisms that recognize the same ligand and employ the same regulatory mechanism. Using the response curve as a basis of comparison, these mutations may be neutral or shift the response curve in line with modulation of single or multiple parameters. Identifying which parameters are modulated will provide insights into how accessible each parameter is to random point mutations and how evolution effectively tunes the response curve through parameter modulation. Advances in our understanding of the biological utilization of natural riboswitches will enable researchers to better define regulatory niches in a biological system and more effectively design synthetic riboswitches to match these niches. Beyond riboswitch design and implementation, insights into the fine-tuning of natural regulatory components and networks will enable the construction of biological networks that reliably control systems-level functions.

## Materials and Methods

### Mathematical modeling

All modeling assumptions and methods are fully described in Text S2. Briefly, time-dependent differential equations were generated using mass action kinetics to describe each mechanistic step in the simplified molecular descriptions of riboswitch function for translational repression, transcriptional termination, and mRNA degradation. The resulting equations were simplified by assuming steady-state conditions. Relevant tuning properties were identified based on the impact of model parameters on the response curve descriptors, including dynamic range (η) defined as the difference between high and low protein levels, ligand concentration to induce a half-maximal response (EC_50_), basal protein levels (P(L = 0)), and maximum-ligand protein levels (P(L→L' or ∞)).

### Plasmid construction

pSAL8.3 served as the base plasmid for all experimental studies [18]. A theophylline-dependent synthetic riboswitch functioning through translational repression resides between the upstream P_tac1_ promoter and the downstream Tn10-β-Galactosidase fusion gene. Mutant sequences were cloned into the unique KpnI and HindIII restriction sites located directly upstream of the riboswitch and approximately 200 nucleotides into the fusion gene coding region. Primers harboring mutant sequences (Table S1) and a 5′ KpnI site were used to PCR amplify the 5′ untranslated region extending through the HindIII restriction site. The resulting PCR product was digested with KpnI/HindIII, ligated into pSAL8.3 digested with the same restriction enzymes, and transformed into *Escherichia coli* strain DH10B. Assembled plasmid constructs were verified by sequencing (Laragen). All molecular biology reagents and enzymes were obtained from New England Biolabs.

### β-Galactosidase activity assay

β-Galactosidase assays were conducted using *E. coli* DH10B cells harboring the pSAL8.3 plasmid mutants based on modifications to previously described protocols [18],[45]. Cells harboring each construct were grown overnight in Luria-Bertani (LB) broth supplemented with 50 µg/ml ampicillin. Overnight cultures were back-diluted into three separate wells containing 500 µl LB broth with 50 µg/ml ampicillin and the appropriate concentration of theophylline and grown at 37°C for 3 hrs with shaking at 210 RPM. Approximately 3 µl of the overnight culture was added to each well. Following the 3-hr incubation with shaking, optical density was recorded by transferring 175 µl into a 96-well microplate with a µClear bottom (Greiner) and measuring on a Safire fluorescence plate reader (Tecan). Cells were lysed by mixing 20 µl of culture with 80 µl permeabilization solution (100 mM Na_2_HPO_4_, 20 mM KCl, 2 mM MgSO_4_, 0.6 mg/ml CTAB, 0.4 mg/ml sodium deoxycholate, and 5.4 µl/ml β-mercaptoethanol) and mixed at room temperature for approximately 10 min. In a fresh 96-well microplate, 25 µl of the lysed culture was mixed with 150 µl substrate solution (60 mM Na_2_HPO_4_, 40 mM NaH_2_PO_4_, 1 mg/ml ONPG, and 5.4 µl/ml β-mercaptoethanol). ONPG hydrolysis was stopped with the addition of 75 µl 1 M Na_2_CO_3_ when a faint yellow color was observed. Absorbance at 420 nm was then measured on the fluorescence plate reader and protein levels were calculated in Miller Units (MU):
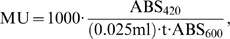
(8)where t is in minutes and absorbance values reflect the difference between each sample and blank media. The MU value of cells carrying a blank plasmid was also subtracted from each sample measurement.

## Supporting Information

Figure S1 Detailed molecular description of riboswitch function. Molecular descriptions shown for riboswitches functioning through (A) translational repression, (B) transcriptional termination, and (C) mRNA destabilization. All riboswitches can reversibly switch between conformations A and B that display different regulatory activities or different rates of degradation. Conformation B contains a formed aptamer that can reversibly bind ligand (L). Transcription is represented as two discrete steps designated by the subscripts I and II, although the model can be altered to include more or less steps. Riboswitches at each step may switch between conformations or reversibly bind ligand depending on the transcribed sequence. The lag between steps is captured by the rate constant k_E_. Once the full riboswitch sequence is transcribed, the riboswitch is susceptible to the regulatory mechanism that controls protein (P) production. Green arrows designate mRNA synthesis with biased transcriptional folding, red arrows designate species degradation, and blue arrows designate translation that is proportional to mRNA levels. Under transcriptional termination (B), riboswitches effectively choose between termination to form a truncated product (T) and extension to form the full-length mRNA (M). To ensure both conformations make the decision with the same frequency, we designated a rate constant k_M_ equal to the sum of the rate constants for extension and termination for either conformation (k_M_ = k_MA_+k_TA_ = k_MB_+k_TB_). We assumed that the riboswitch sequence immediately prior to termination or extension cannot undergo degradation.
(3.60 MB TIF)

Figure S2 Thermodynamically-driven riboswitches exhibiting OFF behavior display similar tuning properties to riboswitches exhibiting ON behavior. K_1_ is the conformational partitioning constant (k_1_′/k_1_) and K_2_ is the aptamer association constant (k_2_/k_2_′). (A) K_1_ affects both basal levels and EC_50_. (B) K_2_ only affects EC_50_. (C) Biased conformational partitioning toward B maximizes the dynamic range at the cost of an increased EC_50_. Parameter values for red response curves are identical to those reported in Figure 2, except K_A_ = 10^−2^/s; K_B_ = 10^−3^/s.
(2.01 MB TIF)

Figure S3 The mechanism-specific regulatory activities dictate differential tuning properties for thermodynamically-driven riboswitches. (A) Modulation of K_A_ and K_B_ affects the maximum dynamic range (η) for ON and OFF behaviors. K_A_ and K_B_ can be independently modulated for riboswitches functioning through translational repression and transcriptional termination. Parameter values for red curve in (A): K_A_ = 10^−3^/s; K_B_ = 10^−2^/s; k_f_ = 6*10^−12^ M/s; k_dP_ = 10^−3^/s; k_dMA_ = k_dMB_ = 10^−3^/s. Both dynamic range and its dependence on K_1_ change when irreversible rates are modulated, showing different trends for (B) ON and (C) OFF behaviors. The degradation rate constants k_dMA_ and k_dMB_ impact steady-state mRNA levels, thereby influencing the dynamic range. Parameter values for red curves in (B) and (C): K_A_ = K_B_ = 1.4*10^−2^/s; k_f_ = 6*10^−12^ M/s; k_dP_ = 10^−3^/s; k_dMA_ = 6*10^−3^/s and k_dMB_ = 10^−3^/s for ON behavior; k_dMA_ = 10^−3^/s and k_dMB_ = 6*10^−3^/s for OFF behavior.
(1.78 MB TIF)

Figure S4 Distinction between tuning properties for ON and OFF behaviors for riboswitches functioning through mRNA destabilization. Dynamic range (η; A,C) and EC_50_ (B,D) display different dependencies on the dominant mRNA degradation rate constant for ON (k_dMA_; A,B) and OFF (k_dMB_; C,D) behaviors. Biased transcriptional folding significantly affects riboswitches displaying ON behavior. Riboswitches displaying OFF behavior show a negligible dependence on transcriptional folding (C, inset) for the selected parameter values. Parameter values: k_1_ = 5*10^−3^; k_1_′ = 2*10^−1^; k_2_ = 10^6^/M*s; k_2_′ = 10^−3^/s; k_P_ = 10^−3^/s; k_f_ = 10^−11^ M/s; k_dP_ = 10^−3^/s; k_dMA_ = 10^−4^/s for OFF behavior; k_dMB_ = 10^−4^/s for ON behavior.
(1.78 MB TIF)

Figure S5 The dynamic range difference and ratio exhibit qualitatively similar tuning properties. Model predictions for the dynamic range difference (η_D_, A) and ratio (η_R_, B) when subjected to a ligand concentration upper limit (L'). In the absence of a ligand concentration upper limit, the dynamic range converges on a maximum (η_max_). The optimum value of the conformational partitioning constant (K_1_) is higher for the dynamic range ratio as the ratio favors lower basal levels. Increasing the aptamer association constant (K_2_) or L' improve the suboptimal dynamic range maximum. Parameter values for the red curves are identical to those reported in Figure 5, and notation is identical to that used in Figure 5B. (C) β-Galactosidase assay results from Figure 6B, where the dynamic range is calculated as the ratio of β-Galactosidase levels in the presence (filled circle) and absence (empty circle) of 1 mM theophylline. The positive control construct (empty) harbors only the RBS and aptamer basal stem. A slight increase in β-Galactosidase activity was observed in the presence of theophylline for the control construct. The experimental data follow the general trends predicted from the model, including the higher optimum K_1_ value for the dynamic range ratio as compared to the dynamic range difference. β-Galactosidase levels are reported in Miller Units (MU). Data represent independent measurements of triplicate samples, where the standard error was below 5% of each mean value.
(2.25 MB TIF)

Table S1 Sequence variants of pSAL8.3 and associated β-Galactosidase levels reported in Miller Units (MU). Database # is included for plasmid requests. The start codon is shown in green and point mutations are shown in blue. To generate each variant, the 5′ end of the β-Galactosidase coding region was amplified and cloned into KpnI/HindIII of pSAL8.3 using primers 5′-AATAGGTACC-[Seq]-TGCGAACTC-3′ and 5′-CGACGGGATCGATCCCCCC-3′, where [Seq] is the designated sequence in the table.
(0.09 MB PDF)

Text S1 Alternative definitions for dynamic range.
(0.04 MB DOC)

Text S2 Derivation of mathematical models.
(0.22 MB DOC)

## References

[1] Acar M, Mettetal JT, van Oudenaarden A (2008). Stochastic switching as a survival strategy in fluctuating environments.. Nat Genet.

[2] Bennett MR, Pang WL, Ostroff NA, Baumgartner BL, Nayak S (2008). Metabolic gene regulation in a dynamically changing environment.. Nature.

[3] Zaslaver A, Mayo AE, Rosenberg R, Bashkin P, Sberro H (2004). Just-in-time transcription program in metabolic pathways.. Nat Genet.

[4] Dekel E, Alon U (2005). Optimality and evolutionary tuning of the expression level of a protein.. Nature.

[5] Suel GM, Kulkarni RP, Dworkin J, Garcia-Ojalvo J, Elowitz MB (2007). Tunability and noise dependence in differentiation dynamics.. Science.

[6] Yokobayashi Y, Weiss R, Arnold FH (2002). Directed evolution of a genetic circuit.. Proc Natl Acad Sci U S A.

[7] Hao N, Nayak S, Behar M, Shanks RH, Nagiec MJ (2008). Regulation of cell signaling dynamics by the protein kinase-scaffold Ste5.. Mol Cell.

[8] Levchenko A, Bruck J, Sternberg PW (2000). Scaffold proteins may biphasically affect the levels of mitogen-activated protein kinase signaling and reduce its threshold properties.. Proc Natl Acad Sci U S A.

[9] Barrick JE, Breaker RR (2007). The distributions, mechanisms, and structures of metabolite-binding riboswitches.. Genome Biol.

[10] Winkler WC (2005). Riboswitches and the role of noncoding RNAs in bacterial metabolic control.. Curr Opin Chem Biol.

[11] Winkler WC, Nahvi A, Roth A, Collins JA, Breaker RR (2004). Control of gene expression by a natural metabolite-responsive ribozyme.. Nature.

[12] Klein DJ, Ferre-D'Amare AR (2006). Structural basis of glmS ribozyme activation by glucosamine-6-phosphate.. Science.

[13] Suess B, Weigand JE (2008). Engineered riboswitches: Overview, problems and trends.. RNA Biol.

[14] Isaacs FJ, Dwyer DJ, Collins JJ (2006). RNA synthetic biology.. Nat Biotechnol.

[15] Osborne SE, Ellington AD (1997). Nucleic acid selection and the challenge of combinatorial chemistry.. Chem Rev.

[16] Bayer TS, Smolke CD (2005). Programmable ligand-controlled riboregulators of eukaryotic gene expression.. Nat Biotechnol.

[17] Beisel CL, Bayer TS, Hoff KG, Smolke CD (2008). Model-guided design of ligand-regulated RNAi for programmable control of gene expression.. Mol Syst Biol.

[18] Lynch SA, Desai SK, Sajja HK, Gallivan JP (2007). A high-throughput screen for synthetic riboswitches reveals mechanistic insights into their function.. Chem Biol.

[19] Win MN, Smolke CD (2007). A modular and extensible RNA-based gene-regulatory platform for engineering cellular function.. Proc Natl Acad Sci U S A.

[20] Isaacs FJ, Dwyer DJ, Ding C, Pervouchine DD, Cantor CR (2004). Engineered riboregulators enable post-transcriptional control of gene expression.. Nat Biotechnol.

[21] Wickiser JK, Cheah MT, Breaker RR, Crothers DM (2005). The kinetics of ligand binding by an adenine-sensing riboswitch.. Biochemistry.

[22] Wickiser JK, Winkler WC, Breaker RR, Crothers DM (2005). The speed of RNA transcription and metabolite binding kinetics operate an FMN riboswitch.. Mol Cell.

[23] Collins JA, Irnov I, Baker S, Winkler WC (2007). Mechanism of mRNA destabilization by the glmS ribozyme.. Genes Dev.

[24] Breaker RR (2008). Complex riboswitches.. Science.

[25] Rieder R, Lang K, Graber D, Micura R (2007). Ligand-induced folding of the adenosine deaminase A-riboswitch and implications on riboswitch translational control.. ChemBioChem.

[26] Lemay JF, Penedo JC, Tremblay R, Lilley DM, Lafontaine DA (2006). Folding of the adenine riboswitch.. Chem Biol.

[27] An CI, Trinh VB, Yokobayashi Y (2006). Artificial control of gene expression in mammalian cells by modulating RNA interference through aptamer-small molecule interaction.. RNA.

[28] Desai SK, Gallivan JP (2004). Genetic screens and selections for small molecules based on a synthetic riboswitch that activates protein translation.. J Am Chem Soc.

[29] Suess B, Hanson S, Berens C, Fink B, Schroeder R (2003). Conditional gene expression by controlling translation with tetracycline-binding aptamers.. Nucleic Acids Res.

[30] Zimmermann GR, Wick CL, Shields TP, Jenison RD, Pardi A (2000). Molecular interactions and metal binding in the theophylline-binding core of an RNA aptamer.. RNA.

[31] Jenison RD, Gill SC, Pardi A, Polisky B (1994). High-resolution molecular discrimination by RNA.. Science.

[32] Cheah MT, Wachter A, Sudarsan N, Breaker RR (2007). Control of alternative RNA splicing and gene expression by eukaryotic riboswitches.. Nature.

[33] Lang K, Rieder R, Micura R (2007). Ligand-induced folding of the thiM TPP riboswitch investigated by a structure-based fluorescence spectroscopic approach.. Nucleic Acids Res.

[34] Greenleaf WJ, Frieda KL, Foster DA, Woodside MT, Block SM (2008). Direct observation of hierarchical folding in single riboswitch aptamers.. Science.

[35] Li PT, Vieregg J, Tinoco I (2008). How RNA unfolds and refolds.. Annu Rev Biochem.

[36] Woodside MT, Anthony PC, Behnke-Parks WM, Larizadeh K, Herschlag D (2006). Direct measurement of the full, sequence-dependent folding landscape of a nucleic acid.. Science.

[37] Lee TH, Lapidus LJ, Zhao W, Travers KJ, Herschlag D (2007). Measuring the folding transition time of single RNA molecules.. Biophys J.

[38] Al-Hashimi HM, Walter NG (2008). RNA dynamics: it is about time.. Curr Opin Struct Biol.

[39] Mathews DH, Disney MD, Childs JL, Schroeder SJ, Zuker M (2004). Incorporating chemical modification constraints into a dynamic programming algorithm for prediction of RNA secondary structure.. Proc Natl Acad Sci U S A.

[40] Parisien M, Major F (2008). The MC-Fold and MC-Sym pipeline infers RNA structure from sequence data.. Nature.

[41] Danilova LV, Pervouchine DD, Favorov AV, Mironov AA (2006). RNAKinetics: a web server that models secondary structure kinetics of an elongating RNA.. J Bioinform Comput Biol.

[42] Topp S, Gallivan JP (2007). Guiding bacteria with small molecules and RNA.. J Am Chem Soc.

[43] Topp S, Gallivan JP (2008). Random walks to synthetic riboswitches—a high-throughput selection based on cell motility.. ChemBioChem.

[44] Tomsic J, McDaniel BA, Grundy FJ, Henkin TM (2008). Natural variability in S-adenosylmethionine (SAM)-dependent riboswitches: S-box elements in bacillus subtilis exhibit differential sensitivity to SAM In vivo and in vitro.. J Bacteriol.

[45] Zhang X, Bremer H (1995). Control of the Escherichia coli rrnB P1 promoter strength by ppGpp.. J Biol Chem.

[46] Voigt CA, Wolf DM, Arkin AP (2005). The Bacillus subtilis sin operon: an evolvable network motif.. Genetics.

[47] Pan T, Sosnick T (2006). RNA folding during transcription.. Annu Rev Biophys Biomol Struct.

[48] Zarrinkar PP, Wang J, Williamson JR (1996). Slow folding kinetics of RNase P RNA.. RNA.

[49] Zhuang X, Bartley LE, Babcock HP, Russell R, Ha T (2000). A single-molecule study of RNA catalysis and folding.. Science.

[50] Su LJ, Waldsich C, Pyle AM (2005). An obligate intermediate along the slow folding pathway of a group II intron ribozyme.. Nucleic Acids Res.

[51] Kensch O, Connolly BA, Steinhoff HJ, McGregor A, Goody RS (2000). HIV-1 reverse transcriptase-pseudoknot RNA aptamer interaction has a binding affinity in the low picomolar range coupled with high specificity.. J Biol Chem.

[52] Win MN, Klein JS, Smolke CD (2006). Codeine-binding RNA aptamers and rapid determination of their binding constants using a direct coupling surface plasmon resonance assay.. Nucleic Acids Res.

[53] Crothers DM, Cole PE, Hilbers CW, Shulman RG (1974). The molecular mechanism of thermal unfolding of Escherichia coli formylmethionine transfer RNA.. J Mol Biol.

[54] Selinger DW, Saxena RM, Cheung KJ, Church GM, Rosenow C (2003). Global RNA half-life analysis in Escherichia coli reveals positional patterns of transcript degradation.. Genome Res.

[55] Narsai R, Howell KA, Millar AH, O'Toole N, Small I (2007). Genome-wide analysis of mRNA decay rates and their determinants in Arabidopsis thaliana.. Plant Cell.

[56] Bernstein JA, Khodursky AB, Lin PH, Lin-Chao S, Cohen SN (2002). Global analysis of mRNA decay and abundance in Escherichia coli at single-gene resolution using two-color fluorescent DNA microarrays.. Proc Natl Acad Sci U S A.

[57] Leclerc GJ, Leclerc GM, Barredo JC (2002). Real-time RT-PCR analysis of mRNA decay: half-life of Beta-actin mRNA in human leukemia CCRF-CEM and Nalm-6 cell lines.. Cancer Cell Int.

[58] Emilsson GM, Nakamura S, Roth A, Breaker RR (2003). Ribozyme speed limits.. RNA.

[59] Belle A, Tanay A, Bitincka L, Shamir R, O'Shea EK (2006). Quantification of protein half-lives in the budding yeast proteome.. Proc Natl Acad Sci U S A.

[60] Corish P, Tyler-Smith C (1999). Attenuation of green fluorescent protein half-life in mammalian cells.. Protein Eng.

