# Supplementary figures and images for: Design Principles for Riboswitch Function
Source: PLoS Comput Biol. 2009 Apr 17;5(4):e1000363. doi: 10.1371/journal.pcbi.1000363 (PMC2666153; doi:10.1371/journal.pcbi.1000363)

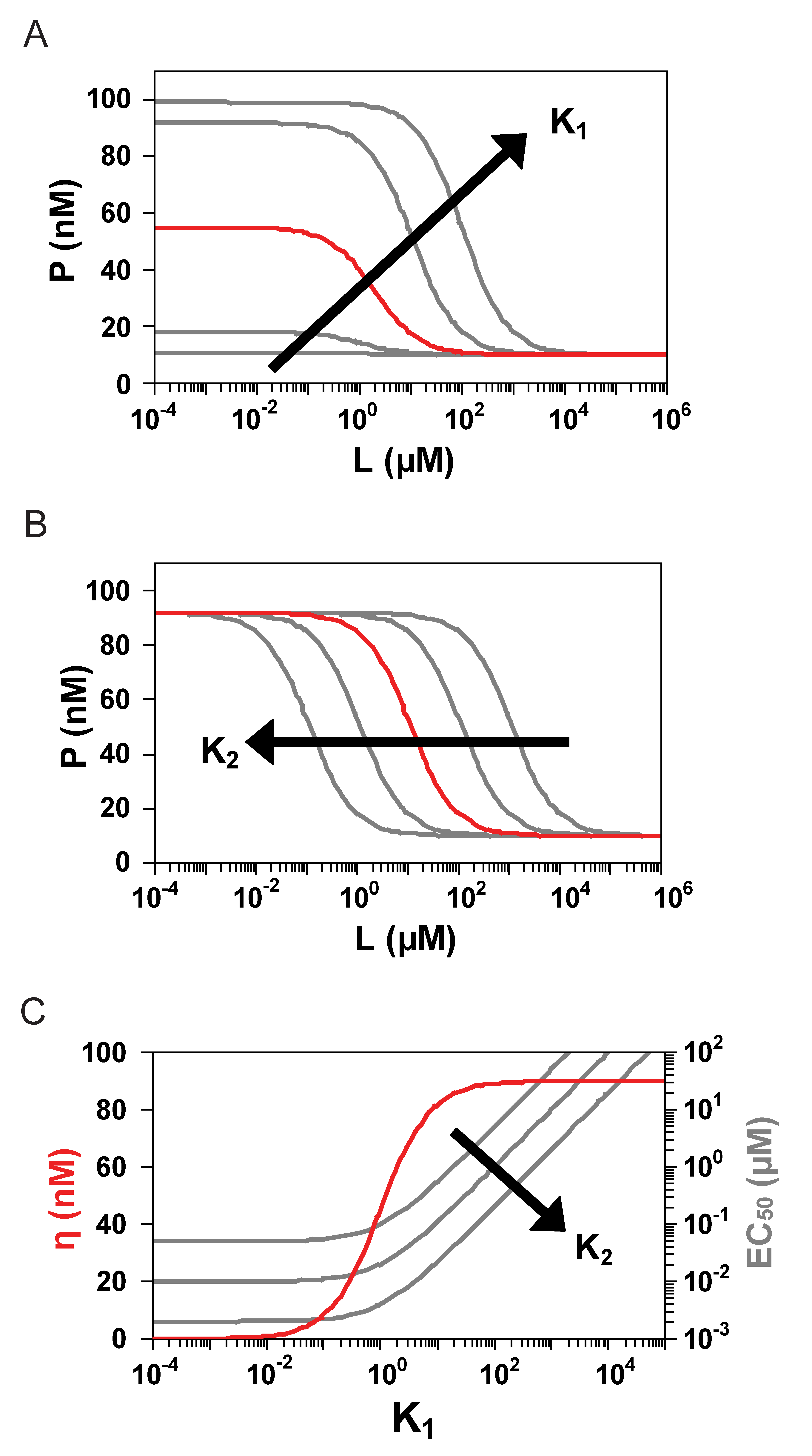

Supplement: Figure S2 — Thermodynamically-driven riboswitches exhibiting OFF behavior display similar tuning properties to riboswitches exhibiting ON behavior. K1 is the conformational partitioning constant (k1′/k1) and K2 is the aptamer association constant (k2/k2′). (A) K1 affects both basal levels and EC50. (B) K2 only affects EC50. (C) Biased conformational partitioning toward B maximizes the dynamic range at the cost of an increased EC50. Parameter values for red response curves are identical to those reported in Figure 2, except KA = 10−2/s; KB = 10−3/s. (2.01 MB TIF) [file pcbi.1000363.s002.tif]

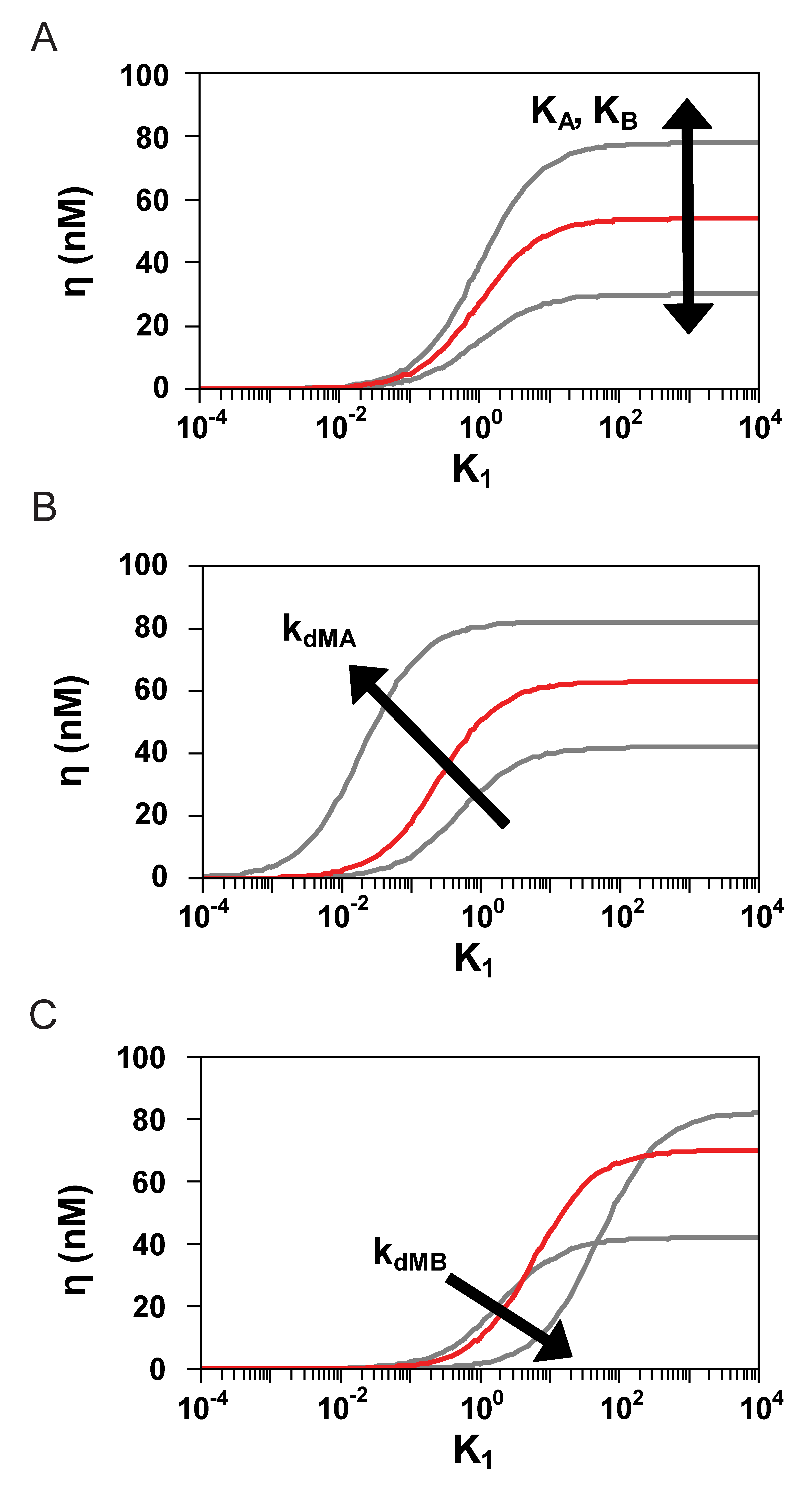

Supplement: Figure S3 — The mechanism-specific regulatory activities dictate differential tuning properties for thermodynamically-driven riboswitches. (A) Modulation of KA and KB affects the maximum dynamic range (η) for ON and OFF behaviors. KA and KB can be independently modulated for riboswitches functioning through translational repression and transcriptional termination. Parameter values for red curve in (A): KA = 10−3/s; KB = 10−2/s; kf = 6*10−12 M/s; kdP = 10−3/s; kdMA = kdMB = 10−3/s. Both dynamic range and its dependence on K1 change when irreversible rates are modulated, showing different trends for (B) ON and (C) OFF behaviors. The degradation rate constants kdMA and kdMB impact steady-state mRNA levels, thereby influencing the dynamic range. Parameter values for red curves in (B) and (C): KA = KB = 1.4*10−2/s; kf = 6*10−12 M/s; kdP = 10−3/s; kdMA = 6*10−3/s and kdMB = 10−3/s for ON behavior; kdMA = 10−3/s and kdMB = 6*10−3/s for OFF behavior. (1.78 MB TIF) [file pcbi.1000363.s003.tif]

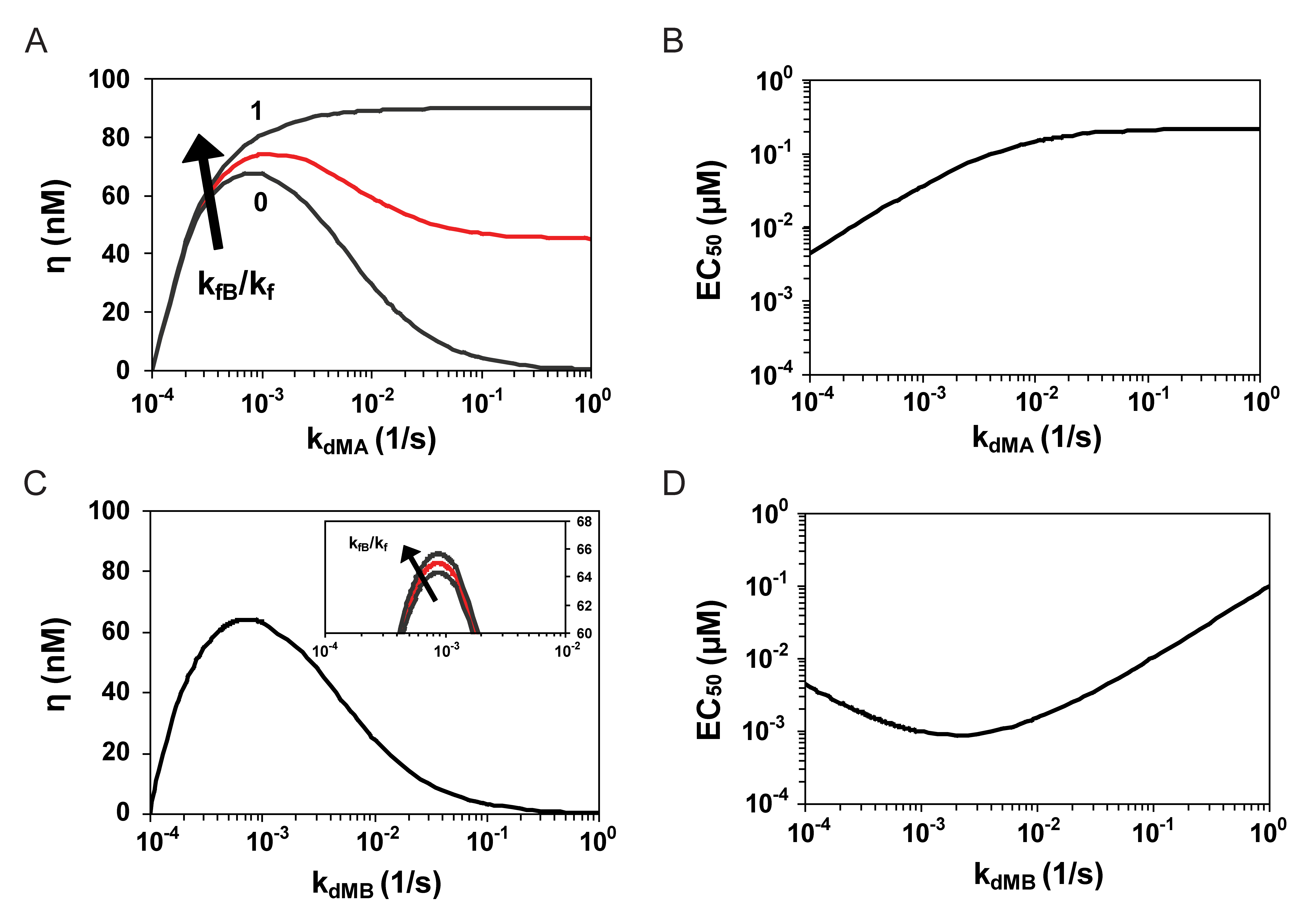

Supplement: Figure S4 — Distinction between tuning properties for ON and OFF behaviors for riboswitches functioning through mRNA destabilization. Dynamic range (η; A,C) and EC50 (B,D) display different dependencies on the dominant mRNA degradation rate constant for ON (kdMA; A,B) and OFF (kdMB; C,D) behaviors. Biased transcriptional folding significantly affects riboswitches displaying ON behavior. Riboswitches displaying OFF behavior show a negligible dependence on transcriptional folding (C, inset) for the selected parameter values. Parameter values: k1 = 5*10−3; k1′ = 2*10−1; k2 = 106/M*s; k2′ = 10−3/s; kP = 10−3/s; kf = 10−11 M/s; kdP = 10−3/s; kdMA = 10−4/s for OFF behavior; kdMB = 10−4/s for ON behavior. (1.78 MB TIF) [file pcbi.1000363.s004.tif]

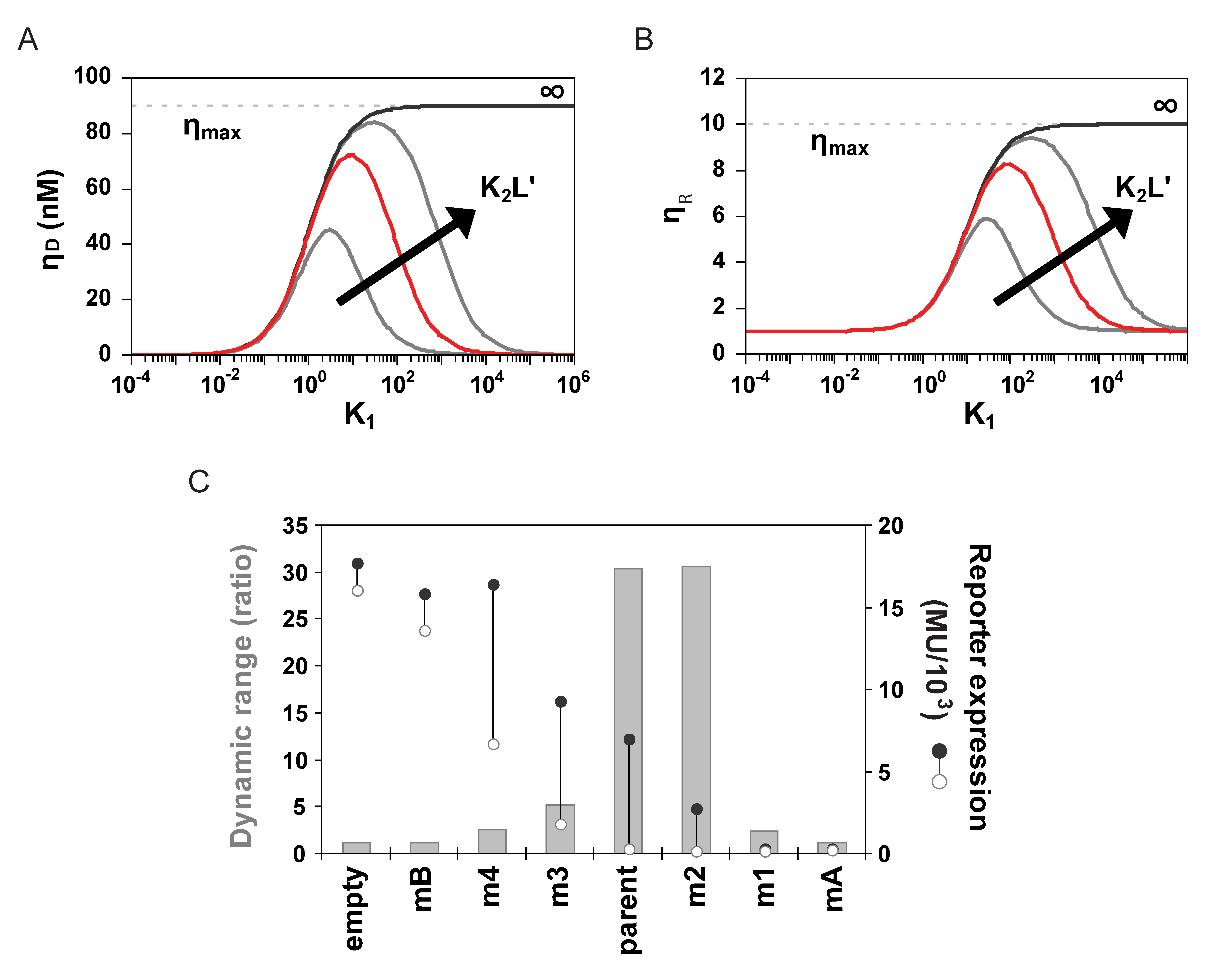

Supplement: Figure S5 — The dynamic range difference and ratio exhibit qualitatively similar tuning properties. Model predictions for the dynamic range difference (ηD, A) and ratio (ηR, B) when subjected to a ligand concentration upper limit (L'). In the absence of a ligand concentration upper limit, the dynamic range converges on a maximum (ηmax). The optimum value of the conformational partitioning constant (K1) is higher for the dynamic range ratio as the ratio favors lower basal levels. Increasing the aptamer association constant (K2) or L' improve the suboptimal dynamic range maximum. Parameter values for the red curves are identical to those reported in Figure 5, and notation is identical to that used in Figure 5B. (C) β-Galactosidase assay results from Figure 6B, where the dynamic range is calculated as the ratio of β-Galactosidase levels in the presence (filled circle) and absence (empty circle) of 1 mM theophylline. The positive control construct (empty) harbors only the RBS and aptamer basal stem. A slight increase in β-Galactosidase activity was observed in the presence of theophylline for the control construct. The experimental data follow the general trends predicted from the model, including the higher optimum K1 value for the dynamic range ratio as compared to the dynamic range difference. β-Galactosidase levels are reported in Miller Units (MU). Data represent independent measurements of triplicate samples, where the standard error was below 5% of each mean value. (2.25 MB TIF) [file pcbi.1000363.s005.tif]
