# Supplementary material for: Design Principles for Riboswitch Function
Source: PLoS Comput Biol. 2009 Apr 17;5(4):e1000363. doi: 10.1371/journal.pcbi.1000363 (PMC2666153; doi:10.1371/journal.pcbi.1000363)
Supplement: Table S1 — Sequence variants of pSAL8.3 and associated β-Galactosidase levels reported in Miller Units (MU). Database # is included for plasmid requests. The start codon is shown in green and point mutations are shown in blue. To generate each variant, the 5′ end of the β-Galactosidase coding region was amplified and cloned into KpnI/HindIII of pSAL8.3 using primers 5′-AATAGGTACC-[Seq]-TGCGAACTC-3′ and 5′-CGACGGGATCGATCCCCCC-3′, where [Seq] is the designated sequence in the table. (0.09 MB PDF) [file pcbi.1000363.s006.pdf]

Table S1 Sequence variants of pSAL8.3 and associated β-Galactosidase levels reported in Miller Units (MU).

Database # is included for plasmid requests. The start codon is shown in green and point mutations are shown in blue. To generate each variant, the 5’ end of the β-Galactosidase coding region was amplified and cloned into KpnI/HindIII of pSAL8.3 using primers 5’-AATAGGTACC-[Seq]-TGCGAACTC-3’ and 5’-CGACGGGATCGATCCCCCC-3’, where [Seq] is the designated sequence in the table.

Name	Sequence	LacZ levels (MU)		Database #
		0 mM	1 mM	
parent	-GGT--GATACCAGCATCGTCTTGATGCCCTTGGCAGC--ACC-AGCTGCAAAGACAACAAG**ATG**	227	6876	pCS1301
m1	-**AA**T--GATACCAGCATCGTCTTGATGCCCTTGGCAGC--A**TT**-AGCTGCAAAGACAACAAG**ATG**	98	234	pCS1315
m2	-**A**GT--GATACCAGCATCGTCTTGATGCCCTTGGCAGC--AC**T**-AGCTGCAAAGACAACAAG**ATG**	87	2653	pCS1314
m3	-GGTC-GATACCAGCATCGTCTTGATGCCCTTGGCAGC-GACC-AGCTGCAAAGACAACAAG**ATG**	1778	9199	pCS1326
m4	-GGTCCGATACCAGCATCGTCTTGATGCCCTTGGCAGCGGACC-AGCTGCAAAGACAACAAG**ATG**	6667	16380	pCS1327
mA	-GGT--**A**ATACCAGCA-CGTCTTGATG**A**CCTT**A**GCAGC--ACC-AGCTGCAAAGACAACAAG**ATG**	170	188	pCS1326
mB	GGGT--GATACCAGCAT**GAAGAGC**ATGCCCTTGGC**TC**C--ACCCAGCTGCAAAGACAACAAG**ATG**	13557	15796	pCS1325
empty	-GGT--GATA-------------------------AGC--ACC-AGCTGCAAAGACAACAAG**ATG**	15980	17661	pCS1324
