# Supplementary material for: Design Principles for Riboswitch Function
Source: PLoS Comput Biol. 2009 Apr 17;5(4):e1000363. doi: 10.1371/journal.pcbi.1000363 (PMC2666153; doi:10.1371/journal.pcbi.1000363)
Supplement: Text S1 — Alternative definitions for dynamic range. (0.04 MB DOC) [file pcbi.1000363.s007.doc]

**ALTERNATIVE DEFINITIONS FOR DYNAMIC RANGE**

The performance of an inducible regulatory system, such as a riboswitch, can be fully defined by a small collection of response curve descriptors: the dynamic range, effective inducer concentration to achieve a half-maximal response (EC_50_), basal or ligand-saturating protein levels, and hill coefficient. Of these descriptors, the dynamic range is the most popular single measure of performance when comparing systems. The dynamic range can be reported as either the ratio of high to low protein levels (η_R_) or the difference between these levels (η_D_). While equally acceptable, one calculation method may be more appropriate than the other depending on the character of the response curve and the performance requirements of the system in which the riboswitch will be integrated. Measuring the performance of a riboswitch by its dynamic range without regard to other descriptors can inappropriately bias the selection of a suitable regulatory system for a given application. For example, a dynamic range ratio of 5 has a very different functional meaning for a system with a basal protein level of 1 molecule per cell than 50 molecules per cell. Dynamic range ratio values are biased to favor minimized basal protein levels, whereas dynamic range difference values are biased to favor larger absolute changes in protein levels.

Both measures of dynamic range are used to address performance requirements for the intended application. In most applications, the inducible regulatory system mediates switching between two phenotypic states determined by expression levels of the regulated genes. The transition between these two states is application-dependent with regards to the regulated gene expression threshold to switch phenotypes and the sensitivity around this threshold. Therefore, the selected inducible regulatory system must allow gene induction or repression across this threshold, requiring basal and ligand-saturating levels outside the range sensitive to transition. Additional restrictions on upper and lower basal and ligand-saturating levels may exist due to the impact of excessively high or low protein levels on cellular fitness and function. Since these factors will be application-specific, the performance properties of the regulatory system will most likely need to be tuned. As discussed in the main text, the kinetics of riboswitch function can be modulated to tune the riboswitch response curve. The relationships between parameters that can be effectively modulated, dynamic range, and other response curve descriptors can be used to tune a riboswitch to meet application-specific performance requirements.

As shown in Table S2 for a thermodynamically-driven riboswitch, η_R_ and η_D_ display differential dependencies on the regulatory activities of conformations A (K_A_) and B (K_B_). Calculation of η_R_ is the same for all riboswitch mechanisms and maintains K_A_/K_B_, such that this value is dimensionless and insensitive to parameter modulations that equally affect K_A_ and K_B_. The drawback to using η_R_ in computational analyses is that the equations for ON and OFF behaviors are not equivalent, such that the predicted tuning properties for η_R_ require designation of either ON or OFF behavior. Specifically, η_R_ is linearly dependent on K_A_/K_B_ for OFF behavior and is maximized for an intermediate value of K_A_/K_B_ for ON behavior.

In contrast, the calculation of η_D_ requires units and the ratio of the irreversible rates between conformations B and A. However, the equations for ON and OFF behaviors are equivalent, such that any elucidated tuning properties are applicable to both behaviors. This property simplifies the computational analyses and facilitates the elucidation of general design principles. We therefore reported dynamic range as the difference between high and low protein levels in the main text. However, we also calculated the tuning properties based on the dynamic range ratio for riboswitches operating in the thermodynamically-driven, kinetically-driven, and non-functional regime. In all cases the qualitative tuning properties were similar for η_D_ and η_R_ (data not shown).

**Table S2.** Comparison of dynamic range calculations for a thermodynamically-driven riboswitch.

Dynamic range is calculated as either the difference between (η_D_) or the ratio of (η_R_) high and low protein levels. Definitions of the model parameters are provided in Figure 2A and Text S2.

| **Riboswitch behavior** | **η_D_** | **η_R_** |
| --- | --- | --- |
| **ON (K_B_ > K_A_)** | $\frac{\text{k}_{\text{f}}}{\text{k}_{\text{dP}}}\frac{\text{K}_{1}}{\text{K}_{1}+\frac{\text{k}_{\text{dMB}}}{\text{k}_{\text{dMA}}}}\left(\frac{\text{K}_{\text{B}}}{\text{k}_{\text{dMB}}}-\frac{\text{K}_{\text{A}}}{\text{k}_{\text{dMA}}}\right)$ | $1+\left(\frac{\text{K}_{\text{B}}}{\text{K}_{\text{A}}}\frac{\text{k}_{\text{dMA}}}{\text{k}_{\text{dMB}}}-1\right)\frac{\text{K}_{1}}{\frac{\text{K}_{\text{B}}}{\text{K}_{\text{A}}}\frac{\text{k}_{\text{dMA}}}{\text{k}_{\text{dMB}}}+\text{K}_{1}}$ |
| **OFF (K_A_ > K_B_)** | $\frac{\text{k}_{\text{f}}}{\text{k}_{\text{dP}}}\frac{\text{K}_{1}}{\text{K}_{1}+\frac{\text{k}_{\text{dMB}}}{\text{k}_{\text{dMA}}}}\left(\frac{\text{K}_{\text{A}}}{\text{k}_{\text{dMA}}}-\frac{\text{K}_{\text{B}}}{\text{k}_{\text{dMB}}}\right)$ | $1+\left(\frac{\text{K}_{\text{A}}}{\text{K}_{\text{B}}}\frac{\text{k}_{\text{dMB}}}{\text{k}_{\text{dMA}}}-1\right)\frac{\text{K}_{1}}{1+\text{K}_{1}}$ |
