# Supplementary material for: Design Principles for Riboswitch Function
Source: PLoS Comput Biol. 2009 Apr 17;5(4):e1000363. doi: 10.1371/journal.pcbi.1000363 (PMC2666153; doi:10.1371/journal.pcbi.1000363)
Supplement: Text S2 — Derivation of mathematical models. (0.22 MB DOC) [file pcbi.1000363.s008.doc]

**DERIVATION OF MATHEMATICAL MODELS**

The mathematical models used to investigate riboswitch performance were derived from molecular descriptions reflecting translational repression, transcriptional termination, and mRNA destabilization (Figure 1B-D). Each description tracks an mRNA encoding the riboswitch and regulated gene(s) from birth to death using kinetic rates to separate molecular species. We assumed that after transcription each riboswitch can reversibly fold into two distinct conformations designated as A and B, which neglects complex folding paths, kinetic traps, and misfolding in order to avoid a cumbersome model. Conformation B includes a formed aptamer such that ligand reversibly binds this conformation to generate a ligand-riboswitch complex (BL). While ligand binding during transcription may contribute to riboswitch performance (Wickis*er et* al, 2005a; Wickis*er et* al, 2005b), we initially neglected this phenomenon and designated independent transcription rate constants for conformations A (k_fA_) and B (k_fB_) to reflect biased transcriptional folding.

For clarity, we include each molecular description from the main text along with an explanation of the associated assumptions. Table S3 contains definitions and base values of all rate constants included in the models.

**Figure 1B.** Molecular description for a riboswitch functioning through translational repression. The riboswitch is transcribed and folds into either conformation A (k_fA_) or B (k_fB_), reversibly switches between conformations (k_1_, k_1_’), and undergoes irreversible degradation (k_dM_) independent of conformation. Conformation B reversibly binds (k_2_) and releases (k_2_’) the cognate ligand (L). The two conformations direct translation dependent on the strength and accessibility of the encoded ribosome binding site (k_PA_, k_PB_). We assumed translation does not affect conformational partitioning or mRNA degradation. Once translated, the protein (P) undergoes degradation (k_dP_).

**Figure 1C.** Molecular description for a riboswitch functioning through transcriptional termination. The riboswitch is transcribed and folds into either conformation A (k_fA_) or B (k_fB_) as an intermediate in the transcription process and reversibly switches between conformations (k_1_, k_1_’). Conformation B reversibly binds (k_2_) and releases (k_2_’) the cognate ligand (L). Both conformations choose between termination (k_TA_, k_TB_) and polymerase extension (k_MA_, k_MB_) at the same rate (k_M_ = k_MA_ + k_TA_ = k_MB_ + k_TB_). The full transcript (M) is translated into protein (k_P_) and undergoes degradation (k_dM_). The truncated transcript (T) undergoes degradation (k_dT_) without contributing to protein production. Once translated, the protein (P) undergoes degradation (k_dP_).

**Figure 1D.** Molecular description for a riboswitch functioning through mRNA destabilization. The riboswitch is transcribed and folds into either conformation A (k_fA_) or B (k_fB_) and reversibly switches between conformations (k_1_, k_1_’). Conformation B reversibly binds (k_2_) and releases (k_2_’) the cognate ligand (L). The two conformations direct translation at the same rate (k_P_) and undergo degradation at different rates (k_dMA_, k_dMB_) based on the regulatory mechanism. We assumed translation does not affect conformational partitioning or mRNA degradation. Once translated, the protein (P) undergoes degradation (k_dP_).

**Table S3.** Rate constants used in all models.

| **Rate constant** | **Description** | **Base value** |
| --- | --- | --- |
| k_f_ | Sum of k_fA_ and k_fB_. Represents total rate of transcription initiation | 10^-11^/s |
| k_fA_ | Rate of appearance of conformation A | 5∙10^-12^/s |
| k_fB_ | Rate of appearance of conformation B | 5∙10^-12^/s |
| k_E_ | Rate of transition from a partially-transcribed riboswitch to a full-length riboswitch | 10^-2^/s |
| k_1_ | Rate of switching from conformation A to conformation B | 10^-1^/s |
| k_1_’ | Rate of switching from conformation B to conformation A | 10^1^/s |
| k_2_ | Rate of ligand binding | 10^6^/M∙s |
| k_2_’ | Rate of ligand release | 10^-1^/s |
| k_M_ | Rate of terminator stem formation | 10^-1^/s |
| k_MA_ | Rate of polymerase extension for conformation A | 9.1∙10^-3^/s |
| k_MB_ | Rate of polymerase extension for conformation B | 9.1∙10^-2^/s |
| k_TA_ | Rate of transcriptional termination for conformation A | 9.1∙10^-2^/s |
| k_TB_ | Rate of transcriptional termination for conformation B | 9.1∙10^-3^/s |
| k_dM_ | Rate of transcript degradation | 10^-3^/s |
| k_dMA_ | Rate of transcript degradation associated with conformation A | 10^-3^/s |
| k_dMB_ | Rate of transcript degradation associated with conformation B | 10^-3^/s |
| k_dT_ | Rate of transcript degradation of the truncated product | 10^-3^/s |
| k_P_ | Rate of translation | 10^-2^/s |
| k_PA_ | Rate of translation for conformation A | 10^-3^/s |
| k_PB_ | Rate of translation for conformation B | 10^-2^/s |
| k_dP_ | Rate of protein degradation | 10^-3^/s |

We generated an expression relating ligand concentration (L) and protein levels (P) for each regulatory mechanism and used these expressions to evaluate riboswitch performance. Ordinary differential equations were generated for each molecular species from the associated molecular description assuming mass action kinetics. Each equation was set equal to zero to evaluate performance under steady-state conditions. A set of performance descriptors (dynamic range, EC_50_, basal levels, and ligand-saturating levels) was then calculated to explore the relationship between model parameters and riboswitch performance. Dynamic range was calculated as the difference between high and low expression levels, although calculation of the dynamic range as a ratio of these two values is equally valid (Text S1). Table S4 contains general equations for the response curve descriptors for each regulatory mechanism and Table S5 contains mechanism-specific parameters.

**Table S4.** General equations for performance descriptors.

Performance descriptors include dynamic range difference (η), EC_50_, basal levels (P(L=0)), and ligand-saturating levels (P(L→∞)). Parameters relate to molecular descriptions in Figure 1B-D. K_1_ is the conformational partitioning constant (K_1_ = k_1_’/k_1_) and K_2_ is the aptamer association constant (K_2_ = k_2_/k_2_’). Mechanism-specific irreversible rate constants k_iA_ and k_iB_, competition ratios γ_1_ and γ_2_, and conformational activities K_A_ and K_B_ are described in Table S5. The absolute value sign reflects the sign change between ON (K_B_ > K_A_) and OFF (K_A_ > K_B_) behaviors.

| **Performance descriptor** | **General equation** |
| --- | --- |
| **η** | $\frac{\text{k}_{\text{f}}}{\text{k}_{\text{dP}}}\cdot\frac{\text{K}_{1}\text{γ}_{1}}{\text{K}_{1}\text{γ}_{1}+\frac{\text{k}_{\text{dMB}}}{\text{k}_{\text{dMA}}}}\left(\frac{\text{k}_{\text{fA}}\text{γ}_{1}+\text{k}_{\text{fB}}}{\text{k}_{\text{f}}}\right)\left\vert\frac{\text{K}_{\text{A}}}{\text{k}_{\text{dMA}}}-\frac{\text{K}_{\text{B}}}{\text{k}_{\text{dMB}}}\right\vert$ |
| **EC_50_** | $\frac{1+\text{K}_{1}\text{γ}_{1}\frac{\text{k}_{\text{dMA}}}{\text{k}_{\text{dMB}}}}{\text{K}_{2}\text{γ}_{2}}$ |
| **P(L=0)** | $\frac{\text{k}_{\text{f}}}{\text{k}_{\text{dP}}}\cdot\left[\frac{\text{k}_{\text{fA}}}{\text{k}_{\text{f}}}\frac{\text{K}_{\text{A}}}{\text{k}_{1}}\text{γ}_{1}+\frac{\text{K}_{\text{A}}\text{K}_{1}\text{γ}_{1}+\text{K}_{\text{B}}}{\text{k}_{\text{dMA}}\text{K}_{1}\text{γ}_{1}+\text{k}_{\text{dMB}}}\left(\frac{\text{k}_{\text{fA}}\text{γ}_{1}+\text{k}_{\text{fB}}}{\text{k}_{\text{f}}}\right)\right]$ |
| **P(L→∞)** | $\frac{\text{k}_{\text{f}}}{\text{k}_{\text{dP}}}\cdot\left[\frac{\text{k}_{\text{fA}}}{\text{k}_{\text{f}}}\frac{\text{K}_{\text{A}}}{\text{k}_{1}}\text{γ}_{1}+\frac{\text{K}_{\text{B}}}{\text{k}_{\text{dMB}}}\left(\frac{\text{k}_{\text{fA}}\text{γ}_{1}+\text{k}_{\text{fB}}}{\text{k}_{\text{f}}}\right)\right]$ |

**Table S5.** Mechanism-specific parameters for translational repression (TR), transcriptional termination (TT), and mRNA destabilization (MD).

k_iA_ and k_iB_ represent the irreversible rate constant for each conformation, K_A_ and K_B_ represent the regulatory activity of each conformation, and γ_1_ and γ_2_ represent the competition between reversible and irreversible rate constants. The letters A and B in each suffix reflect the associated conformation. Parameters relate to molecular descriptions in Figure 1B-D. The irreversible rate constant for transcriptional termination (k_M_) is the same for conformations A and B and is equal to the sum of the rate constants associated with termination (k_TA_, k_TB_) and read-through (k_MA_, k_MB_) such that k_M_ = k_MA_ + k_TA_ = k_MB_ + k_TB_.

| **Model parameter** | **Regulatory Mechanism** | | |
| --- | --- | --- | --- |
|  | **TR** | **TT** | **MD** |
| **k_iA_** | k_dM_ | k_M_ | k_dMA_ |
| **k_iB_** |  |  | k_dMB_ |
| **K_A_** | k_PA_ | $\text{k}_{\text{P}}\frac{\text{k}_{\text{MA}}}{\text{k}_{\text{M}}}$ | k_P_ |
| **K_B_** | k_PB_ | $\text{k}_{\text{P}}\frac{\text{k}_{\text{MB}}}{\text{k}_{\text{M}}}$ |  |
| **γ_1_** | $\frac{\text{k}_{1}}{\text{k}_{1}+\text{k}_{\text{dM}}}$ | $\frac{\text{k}_{1}}{\text{k}_{1}+\text{k}_{\text{M}}}$ | $\frac{\text{k}_{1}}{\text{k}_{1}+\text{k}_{\text{dMA}}}$ |
| **γ_2_** |  |  |  |

The majority of our modeling efforts assumed that ligand binding during transcription was not a contributing factor. For thermodynamically-driven riboswitches, this is a valid assumption since the conformational equilibrium attained in the presence of ligand is not affected by extra time or opportunities for ligand binding. However, as discussed in the main text, restoring function to a non-functional riboswitch requires the contribution of ligand binding during transcription. We focused our investigation of this phenomenon on transcriptional termination, since experimental data have suggested that certain natural riboswitches functioning through this mechanism are non-functional based on our definition. The derived response curve descriptors for the molecular description of a non-functional riboswitch functioning through transcriptional termination (Figure 4A) are presented in Table S6.

**Table S6.** Performance descriptors for a non-functional riboswitch functioning through transcriptional termination.

Performance descriptors include dynamic range difference (η), EC_50_, basal levels (P(L=0)), and ligand-saturating levels (P(L→∞)). Parameters relate to the molecular description in Figure 4A. The absolute value sign reflects the sign change between ON (k_MB_ > k_MA_) and OFF (k_MA_ > k_MB_) behaviors. The rate constant for terminator stem formation (k_M_) is the same for conformations A and B and is equal to the sum of the rate constants associated with termination (k_TA_, k_TB_) and read-through (k_MA_, k_MB_) such that k_M_ = k_MA_ + k_TA_ = k_MB_ + k_TB_.

| **Performance descriptor** | **General equation** |
| --- | --- |
| **Η** |  |
| **EC_50_** |  |
| **P(L=0)** |  |
| **P(L→∞)** | $\frac{\text{k}_{\text{f}}\text{k}_{\text{P}}}{\text{k}_{\text{dP}}\text{k}_{\text{dM}}}\cdot\frac{\text{k}_{\text{MB}}}{\text{k}_{\text{M}}}$ |

The above analyses assumed that the ligand concentration can saturate the response curve. However, a practical upper limit to the ligand concentration (L’) exists for any regulatory system. In the main text we discussed the implications of setting a ligand concentration upper limit for thermodynamically-driven riboswitches on the observed tuning properties. All response curve descriptors were evaluated under conditions in which the upper limit to the ligand concentration does not saturate the response curve. The associated equations are presented in Table S7.

**Table S7.** Performance descriptors for a thermodynamically-driven riboswitch subjected to a ligand concentration upper limit (L’).

The general thermodynamically-driven riboswitch was used as the basis for these calculations. Parameters relate to the molecular description in Figure 2A. Dynamic range (η) and the apparent EC_50_ (EC_50_^APP^) reflect a restricted ligand concentration range between 0 and L’.

| **Performance descriptor** | **General equation** |
| --- | --- |
| **Η** |  |
| **EC_50_^APP^** |  |

**REFERENCES**

Wickiser JK, Cheah MT, Breaker RR, Crothers DM (2005a) The kinetics of ligand binding by an adenine-sensing riboswitch. *Biochemistry* **44:** 13404-13414.

Wickiser JK, Winkler WC, Breaker RR, Crothers DM (2005b) The speed of RNA transcription and metabolite binding kinetics operate an FMN riboswitch. *Mol Cell* **18:** 49-60.
